# New species, new records, and common species of *Diderma* (Physarales, *Didymiaceae*) from China

**DOI:** 10.1128/spectrum.01265-24

**Published:** 2024-08-20

**Authors:** Xuefei Li, Yonglan Tuo, Jiajun Hu, You Li, Dan Dai, Frederick Leo Sossah, Jiajia Wang, Yanfang Guo, Shuyan Liu, Haixia Ma, Bo Zhang, Xiao Li, Yu Li

**Affiliations:** 1 Joint International Research Laboratory of Modern Agricultural Technology, Ministry of Education, Jilin Agricultural University, Changchun, Jilin, China; 2 Engineering Research Center of Chinese Ministry of Education for Edible and Medicinal Fungi, Jilin Agricultural University, Changchun, Jilin, China; 3 College of Mycology, Jilin Agricultural University, Changchun, Jilin, China; 4 School of Life Science, Zhejiang Normal University, Jinhua, Zhejiang, China; 5 Institute of Agricultural Applied Microbiology, Jiangxi Academy of Agricultural Sciences, Nanchang, Jiangxi, China; 6 Council for Scientific and Industrial Research (CSIR), Oil Palm Research Institute, Coconut Research Programme, Sekondi, Ghana; 7 Westerdijk Fungal Biodiversity Institute, Utrecht, The Netherlands; 8 Institute of Tropical Bioscience and Biotechnology, Chinese Academy of Tropical Agricultural Sciences, Haikou, Hainan, China; Agroscope, Nyon, Switzerland

**Keywords:** *Diderma*, distribution, morphology, myxomycetes, new species, phylogenetic, taxonomy

## Abstract

Myxomycetes are fungus-like organisms that play a significant role in ecological processes, however, their taxonomic diversity and distribution in China are poorly understood. *Diderma* is an important genus within the Class Myxogastria that has received little attention in China. This study provides new insights into the geographic range of *Diderma* species in China and identifies previously unreported and newly recorded species. Our results reveal that the geographic distribution of *Diderma* species in China is more diverse than previously thought, with four previously unreported species found in Liaoning, Hubei, Sichuan, and Gansu provinces. In addition, we describe five new *Diderma* species that are distinct from previously known species, namely *Diderma annuliferum*, *Diderma gansuense*, *Diderma roseum*, *Diderma jilinense*, and *Diderma flexocapillitium*. We identified these species using a combination of morphological characterization, DNA sequencing, and phylogenetic analysis. Our findings have important implications for understanding Myxomycete biodiversity in China and can inform future research on the ecology, biogeography, and evolution of these fascinating organisms. Specifically, our study highlights the need for continued exploration of underrepresented areas to gain a more comprehensive understanding of the diversity and distribution of Myxomycetes in China.

**IMPORTANCE:**

The discovery of five new *Diderma* species and the revelation of their diverse distribution expand our understanding of Myxomycete diversity and provide a foundation for future studies on the ecology and biogeography of these organisms. These findings contribute to our knowledge of microbial diversity and have practical implications for conserving underrepresented areas and maintaining healthy ecosystems.

## INTRODUCTION

Myxomycetes are a unique group of small eukaryotic organisms that are found in almost all terrestrial ecosystems and are significant to biodiversity (1–6). Among the many genera of myxomycetes, the genus *Diderma* Pers. is particularly important and abundant. The study of this genus has a long history, dating back to its establishment by Persoon in 1794, with *D. globosum* Pers. as the type specimen (7). A classification system is significant for understanding the relationships between different species and genera within the myxomycetes group. It provides a framework for organizing and studying these organisms and can help researchers make connections between different species and better understand their ecological and evolutionary histories. However, the taxonomic status of the genus *Diderma* has been a source of controversy over time.

Rostafinski, considered the founder of myxomycetes taxonomy, placed the genus *Diderma* in the family *Didymiaceae* in 1873 (8–10). However, Lister later assigned it to the family *Physaraceae* (11). In 1969, Martin and Alexopoulos proposed a classification system that once again placed *Diderma* in the family *Didymiaceae*, which became the accepted classification for many years (12). More recently, Leontyev et al. (13) proposed a revised classification system that placed myxomycetes into two subclasses, Lucisporomycetidae and Columellomycetidae. In this system, *Diderma* is placed within the Family *Didymiaceae*, Order Physarales, Superorder Stemonitidia, Subclass Columellomycetidae, and Class Myxomycetes. In recent years, research on myxomycetes has been expanded, leading to an increase in the number of identified *Diderma* species and a broader distribution of these species. As of 2005–2023, the genus *Diderma* has been reported to contain a total of 87 species worldwide (2–6, 14–19), with 30 of these species found in China (which makes up 34.48% of the world’s reports) (15, 16, 18, 19).

The main characteristic of the genus *Diderma* is that the sporophores have irregularly shaped lime granules that are distributed differently depending on the species. Most species are sessile and have a specific texture in their peridium, which can be either brittle and calcareous or ductile and cartilaginous (2–6, 8, 15–19). They prefer to grow in moist and shady environments and obtain nutrients for growth from substrates like dead leaves, mosses, and decaying stumps. They can thrive in various vegetation types and environments, but they have a preference for these conditions.

The genus *Diderma* has not been extensively studied in China, resulting in limited knowledge about its species diversity. However, in recent years, several *Diderma* species have been reported in various regions of China (2–6, 14–19). In this study, we used morphological and phylogenetic analyses of collected specimens to identify and propose five new species (*Diderma annuliferum*, *Diderma gansuense*, *Diderma roseum*, *Diderma jilinense*, and *Diderma flexocapillitium*). Additionally, we included descriptions and illustrations of four newly recorded species from Liaoning, Hubei, Sichuan, and Gansu provinces. The discovery of these species expands our knowledge of the diversity of the genus *Diderma* in China. To further explore the species diversity of *Diderma* in China, we conducted identification, reexamination, and literature research based on recent collections and herbarium specimens at the Herbarium of Mycology of the Jilin Agricultural University (HMJAU), summarizing a total of 35 *Diderma* species key of the genus *Diderma* recorded from China (Table 1) (14–18).

**TABLE 1:** Taxa and GenBank accession numbers used in phylogenetic analysis

Scientific name	Voucher/specimen numbers	GenBank accession numbers			Reference
		nSSU	EF-1α	COI	
*Badhamia melanospora*	MA-Fungi 81706	KC758975	MG963469		(20)
*B. melanospora*	MA-Fungi 88015	MF352450	MF352496		(21)
*B. melanospora*	MA-Fungi 64653	KC759019	MG963468		(20)
*B. melanospora*	MA-Fungi 80423	MF352448	MF352494		(21)
*Diderma alpinum*	sc30044	MN595463	MN596920		(22)
*D. alpinum*	YK385	MN595608	OP616431	OP616538	(23)
***D. annuliferum***	**HMJAU M20001-1**	**PP165419**	**PP178586**		**This study**
***D. annuliferum***	**HMJAU M20001-2**	**PP165420**	**PP178587**		**This study**
***D. cingulatum***	**HMJAU M20004-1**	**PP165426**	**PP178589**		**This study**
***D. cingulatum***	**HMJAU M20004-2**	**PP165427**	**PP178590**	**PP261312**	**This study**
***D. cingulatum***	**HMJAU M20005**	**PP165428**	**PP178591**	**PP261316**	**This study**
***D. cingulatum***	**HMJAU M20006**	**PP165429**	**PP178592**	**PP261317**	**This study**
***D. cingulatum***	**HMJAU M20007**	**PP165430**	**PP178593 **	**PP261318**	**This study**
***D. cor-rubrum***	**HMJAU M20010-1**	**PP165435**			**This study**
***D. cor-rubrum***	**HMJAU M20010-2**	**PP165436**			**This study**
***D. cor-rubrum***	**HMJAU M20011**	**PP165437**	**PP178598**	**PP261319**	**This study**
***D. cor-rubrum***	**HMJAU M20012**	**PP165438 **	**PP178599**		**This study**
*D. cor-rubrum*	MYX11340	OP621217	OP616430	OP616541	(23)
*D. cor-rubrum*	LE302473	OP621216	OP616429	OP616540	(23)
***D. crustaceum***	**HMJAU M20008-1**	**PP165431**	**PP178594**		**This study**
***D. crustaceum***	**HMJAU M20008-2**	**PP165432**	**PP178595**		**This study**
***D. crustaceum***	**HMJAU M20009-1**	**PP165433**	**PP178596**		**This study**
***D. crustaceum***	**HMJAU M20009-2**	**PP165434**	**PP178597**		**This study**
*D. chondrioderma*	MYX439	KM977850	MK555282		(24)
*D. deplanatum*	MYX440	KM977851	MK555283		(24)
***D. effusum***	**HMJAU M20014-1**	**PP165441**	**PP178601**		**This study**
***D. effusum***	**HMJAU M20014-2**	**PP165442**	**PP178602**		**This study**
***D. effusum***	**HMJAU M20015**	**PP165443**	**PP178603**	**PP261320**	**This study**
*D. effusum*	MYX7994	MZ604987	MZ605416	OP616543	(25)
*D. globosum* var. *europaeum*	MA-Fungi 73109	MG963656	MG963487		(20)
*D. globosum* var. *europaeum*	MA-Fungi 73112	MG963657	MG963488		(20)
***D. flexocapillitium***	**HMJAU M20019-1**	**PP165449**			**This study**
***D. flexocapillitium***	**HMJAU M20019-2**	**PP165450**			**This study**
***D. flexocapillitium***	**HMJAU M20020**	**PP165451**	**PP178608**		**This study**
***D. flexocapillitium***	**HMJAU M20021**	**PP165452**	**PP178609**		**This study**
***D. flexocapillitium***	**HMJAU M20022**	**PP165453**	**PP178610**		**This study**
*D. floriforme*	MdH1810007	OM339175			(26)
*D. floriforme*	MYX18793	OP621222	OP616432	OP616547	(23)
***D. gansuense***	**HMJAU M20013-1**	**PP165439**	**PP178600**		**This study**
***D. gansuense***	**HMJAU M20013-2**	**PP165440**			**This study**
*D. globosum*	LE325799	MZ604992	MZ605421	OP616551	(25)
*D. globosum*	LE325800	MZ604993	MZ605422	OP616552	(25)
***D. hemisphaericum***	**HMJAU M20017-1**	**PP165445**			**This study**
***D. hemisphaericum***	**HMJAU M20017-2**	**PP165446**	**PP178605**		**This study**
*D. hemisphaericum*	MA-Fungi 63960	MG963659	MG963490		(20)
*D. hemisphaericum*	MA-Fungi 90974	MG963660	MG963491		(10)
***D. jilinense***	**HMJAU M20042**	**PP165482**	**PP178627**	**PP261327**	**This study**
***D. jilinense***	**HMJAU M20043**	**PP165483**	**PP178628**		**This study**
***D. liaoningensis***	**HMJAU M20018-1**	**PP165447**	**PP178606**		**This study**
***D. liaoningensis***	**HMJAU M20018-2**	**PP165448 **	**PP178607**		**This study**
*D. meyerae*	LE284739	KU198050	KU198109		unpublished
*D. meyerae*	sc30279	MN595488	MN596919		(22)
*D. niveum*	MA-Fungi 78779	MW240312	MW240041		(20)
*D. niveum*	MA-Fungi 78780	MW240313			(20)
*D. pseudotestaceum*	LE291396	KJ659866	KJ676604		(27)
*D. pseudotestaceum*	LE291397	KJ659867	KJ676605		(27)
*D. pseudotestaceum*	LE291398	KJ659864	KJ676602		(27)
*Diderma radiatum*	MYX9867	MZ604983	MZ605412	OP616561	(23, 25)
*D. radiatum*	MYX9071	MK838444	MZ605410	OP616560	(23, 25)
***D. roanense***	**HMJAU M20002-1**	**PP165421**		**PP261315**	**This study**
***D. roanense***	**HMJAU M20002-2**	**PP165422**			**This study**
***D. roanense***	**HMJAU 8877**	**PP165423**			**This study**
***D. roanense***	**HMJAU M20003-1**	**PP165424**	**PP178588**		**This study**
***D. roanense***	**HMJAU M20003-2**	**PP165425**			**This study**
***D. roseum***	**HMJAU M20044**	**PP165484**	**PP178629**	**PP261328**	**This study**
***D. roseum***	**HMJAU M20045**	**PP165485**	**PP178630**		**This study**
***D. roseum***	**HMJAU M20046**	**PP165486**	**PP178631**		**This study**
***D. roseum***	**HMJAU M20047**	**PP165487**	**PP178632**		**This study**
***D. roseum***	**HMJAU M20048**	**PP165488**	**PP178633**		**This study**
***D. roseum***	**HMJAU M20049**	**PP165489**	**PP178634**	**PP261329**	**This study**
***D. saundersii***	**HMJAU M20027-1**	**PP165461**	**PP178616**	**PP261323**	**This study**
***D. saundersii***	**HMJAU M20027-2**	**PP165462**	**PP178617**		**This study**
***D. testaceum***	**HMJAU M20029-1**	**PP165463**	**PP178618**		**This study**
***D. testaceum***	**HMJAU M20029-2**	**PP165464**	**PP178619**		**This study**
***D. testaceum***	**HMJAU M20030**	**PP165465**	**PP178620**		**This study**
***D. testaceum***	**HMJAU M20031**	**PP165466**	**PP178621**		**This study**
*D. velutinum*	LE318752	MH714785	MH717084	MH717086	(28)
*D. velutinum*	LE318753	MH714786	MH717085	MH717087	(28)
***D. verrucocapillitia***	**HMJAU M20032-1**	**PP165467**	**PP178622**		**This study**
***D. verrucocapillitia***	**HMJAU M20032-2**	**PP165468**	**PP178623**		**This study**
*Didymium dubium*	MA-Fungi 63904	MW240326	MW240058		(20)
*D. dubium*	MA-Fungi 80036	MW240327	MW240059		(20)
*D. dubium*	MA-Fungi 80492	MG662512	MW240060		(20)
*D. dubium*	K7	AM231294			(29)
*D. melanospermum*	MA-Fungi 91238	MG963668	MG963497		(20)
*D. melanospermum*	MA-Fungi 62790	MG963667	MW240068		(20)
*D. nivicola*	MA-Fungi 90573	MT227090	MT230925		(30)
*D. nivicola*	AH19667	MT227019	MT230908		(30)
*D. pseudonivicola*	MA-Fungi 90601	MT227112	MT230931		(30)
*D. pseudonivicola*	MA-Fungi 90587	MT227099	MT230927		(30)
*D. yulii*	HMJAU M3002	MF149871	MK905755		(31)
*D. yulii*	HMJAU M3001	MF149870	MK905754		(31)
*Echinostelium coelocephalum*	ATCC MYA 2964	AY842033	AY643813		(32)
*E. minutum*	ATCC 22345	AY842034	AY643814		(32)
*Enerthenema intermedium*	MM 21635	DQ903688			(33)
*E. melanospermum*	MM 28388	DQ903689			(33)
*E. papillatum*	AMFD141	AY643823			(32)
*Fuligo septica*	MA-Fungi 78118	MW240350	MW240091		(20)
*F. septica*	MA-Fungi 78792	MF352458	MF352504		(21)
*F. septica*	MA-Fungi 78801	MF352459	MF352505		(21)
*Lamproderma aeneum*	MA-Fungi 81947	MW240352	MW240092		(20)
*L. aeneum*	MA-Fungi 86925	MW240353	MW240093		(20)
*L. aeneum*	MA-Fungi 90422	MW240354	MW240094		(20)
*L. ovoideum*	Sc30802	MN595543	MN596918		(22)
*Macbrideola oblonga*	M Schnittler	DQ903682			(33)
*Meriderma aggregatum*	AMFD135	DQ903669			(33)
*M. carestiae* var. *retisporum*	AMFD173	DQ903671			(33)
*M. fuscatum*	MM 24907	DQ903668			(33)
*Nannengaella globulifera*	MA-Fungi 51647	MF352479	MF352528		(21)
*N. globulifera*	MA-Fungi 46711	MW240379			(20)
*N. globulifera*	MYX8635	MW693010	MW701657		(34)
*N. globulifera*	MYX8695	MW693014	MW701661		(34)
*N. mellea*	MA-Fungi 69850	MF352484	MF352534		(21)
*N. mellea*	MA-Fungi 87986	MF352485	MF352535		(21)
*N. mellea*	MA-Fungi 60322	MW240383	MG963528		(20)
*Physarum didermoides*	MA-Fungi 71195	MW240378			(20)
*P. didermoides*	MA-Fungi 57262	MF352488	MF352542		(21)
*P. leucophaeum*	MA-Fungi 49730	MG963686	MG963521		(20)
*P. leucophaeum*	MA-Fungi 78861	MG963688			(20)
*P. leucophaeum*	MA-Fungi 59323	MF352477	MF352526		(21)
*P. nivale*	MA-Fungi 72831	MF352486	MF352536		(21)
*P. nivale*	MA-Fungi 73457	MF352487	MF352537		(21)
*P. nivale*	MA-Fungi 70191	MW240384	MG963529		(20)
*P. nivale*	MA-Fungi 70193	MW240385			(20)
*P. pseudonotabiles* s. lat.	LE255432	LT670439	KF250465		(35)
*P. pseudonotabiles* s. lat.	LE255437	LT670419	KC473813		(35, 36)
*P. pseudonotabiles* s. lat.	LE255703	LT670568	KF250468		(35)
*P. pseudonotabiles* s. lat.	LE284662	LT670428	KF250472		(35)
*P. straminipes*	MA-Fungi 70363	MF352489	MF352543		(21)
*P. straminipes*	MA-Fungi 87865	MW240394			(20)
*P. viride* f. *aurantium*	LE302489	MW693022	MW701670		(34)
*P. viride* s. lat.	LE317322	MW693024	MW701672	OP616654	(23, 34)
*Symphytocarpus impexus*	–	AY230188			(37)

^*a*^ Sequences produced in this study are in bold.

## RESULTS

### Phylogenetic analyses

**Fig 1:**
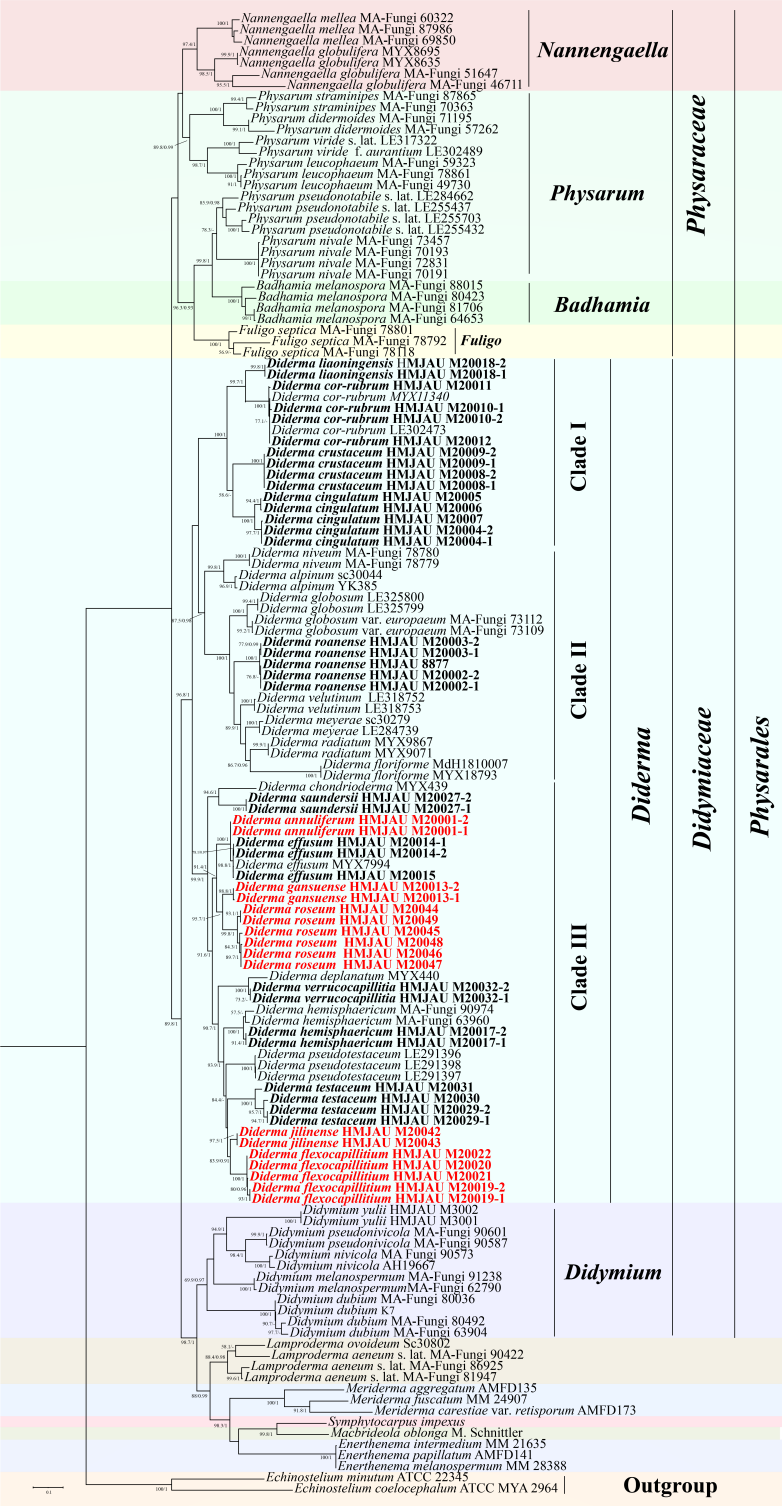
Phylogenetic tree diagram depicting the evolutionary relationships of *Diderma* in myxomycetes. Maximum likelihood phylogenetic tree generated from the nSSU, EF-1α, and COI data set. The values assigned to internal nodes of the tree represent the maximum likelihood (ML) and Bayesian inference (BI) support. Bootstrap values (BP) ≥ 70% from ML analysis and Bayesian posterior probabilities (BPPS) ≥ 0.90 are shown on the branches. Newly sequenced collections are indicated in bold, and the newly discovered species are marked in red.

For the phylogenetic analysis, a tree was constructed using a data set of 263 myxomycetes sequences from 53 species of 12 genera in three orders, including 26 *Diderma* species (Fig. 1). Two sequences from the genus *Echinostelium* de Bary (*E. minutum* de Bary and *E. coelocephalum* T. E. Brooks & H. W. Keller) were used as outgroups. The tree consisted of three main branches: family *Didymiaceae*, family *Physaraceae*, and family *Stemonitaceae*. Among these, 101 sequences were newly generated, comprising 50 nSSU sequences, 40 EF-1α sequences, and 11 COI sequences (Fig. 1). The final data set comprised 10,581 characters, including gaps, with 7,659 characters (including gaps) from SSU, 2,139 characters from EF-1α, and 783 characters from COI.

**Fig 2:**
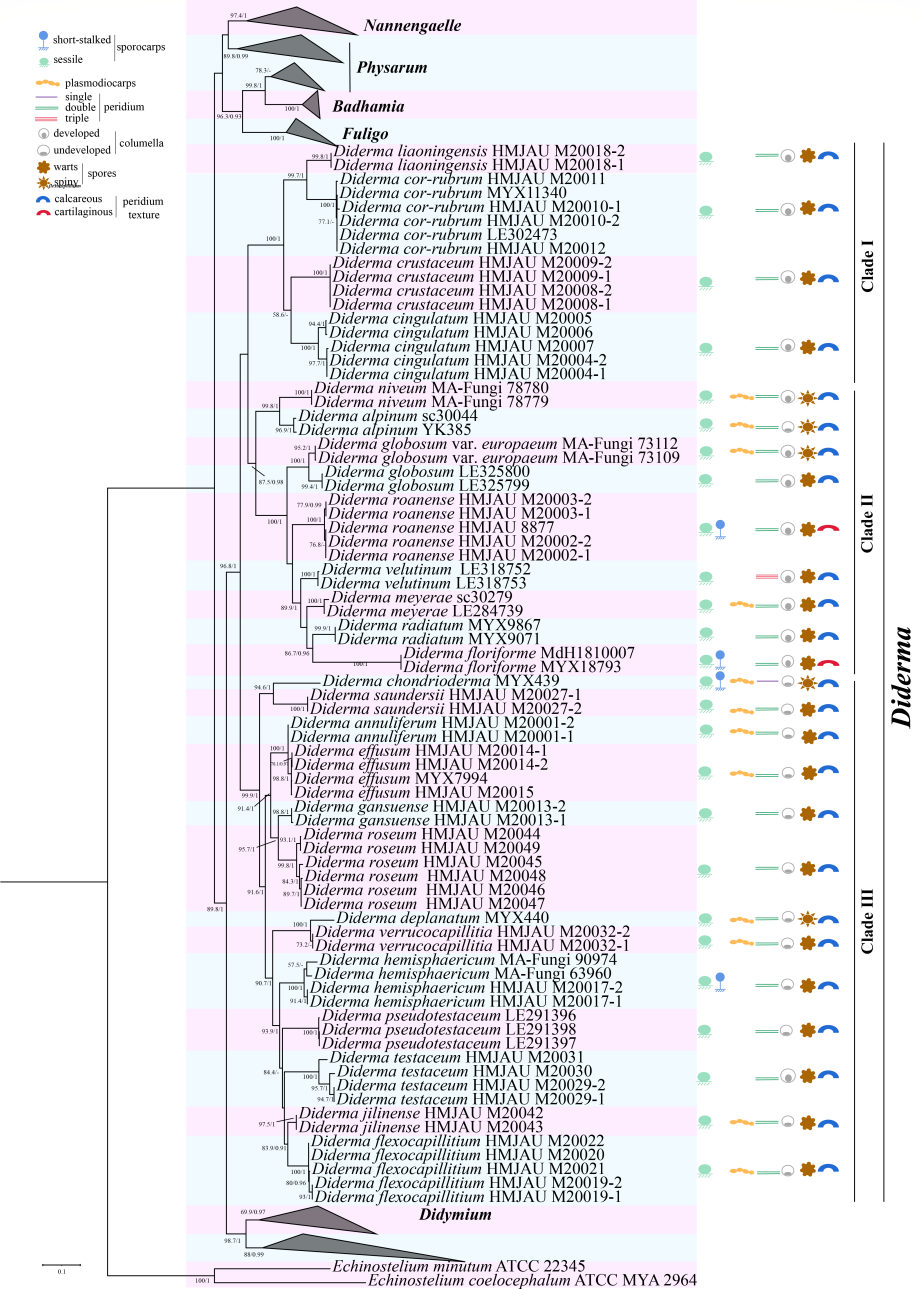
Phylogenetic relationships of *Diderma* inferred from multi-locus (nSSU, EF-1A, and COI) using Bayesian inference and maximum likelihood method (ML tree was shown). BPPS ≥ 0.9 and BP ≥ 60 are indicated in the phylogram with key characteristics listed at the right.

Comparison of the overall topologies between the maximum likelihood (ML) and Bayesian inference (BI) trees indicated nearly identical results across all data sets. Given this congruence, the ML analysis was chosen as the representative phylogeny. The phylogenetic tree within the genus *Diderma* revealed three distinct clades, the species included in Clade, Clade II, and Clade III, we added some new sequences that enriched the number of species in each branch (Fig. 1). In addition, we can observe that 16 isolates formed five lineages distinct from the other species. These five distinct lineages were therefore considered novel species of *Diderma* (Fig. 1), which are consistent with the morphological observations we have made. The phylogram summarizes the key characteristics of *Diderma*, with a detailed list provided on the right side (Fig. 2).

### Taxonomy

**Fig 3:**
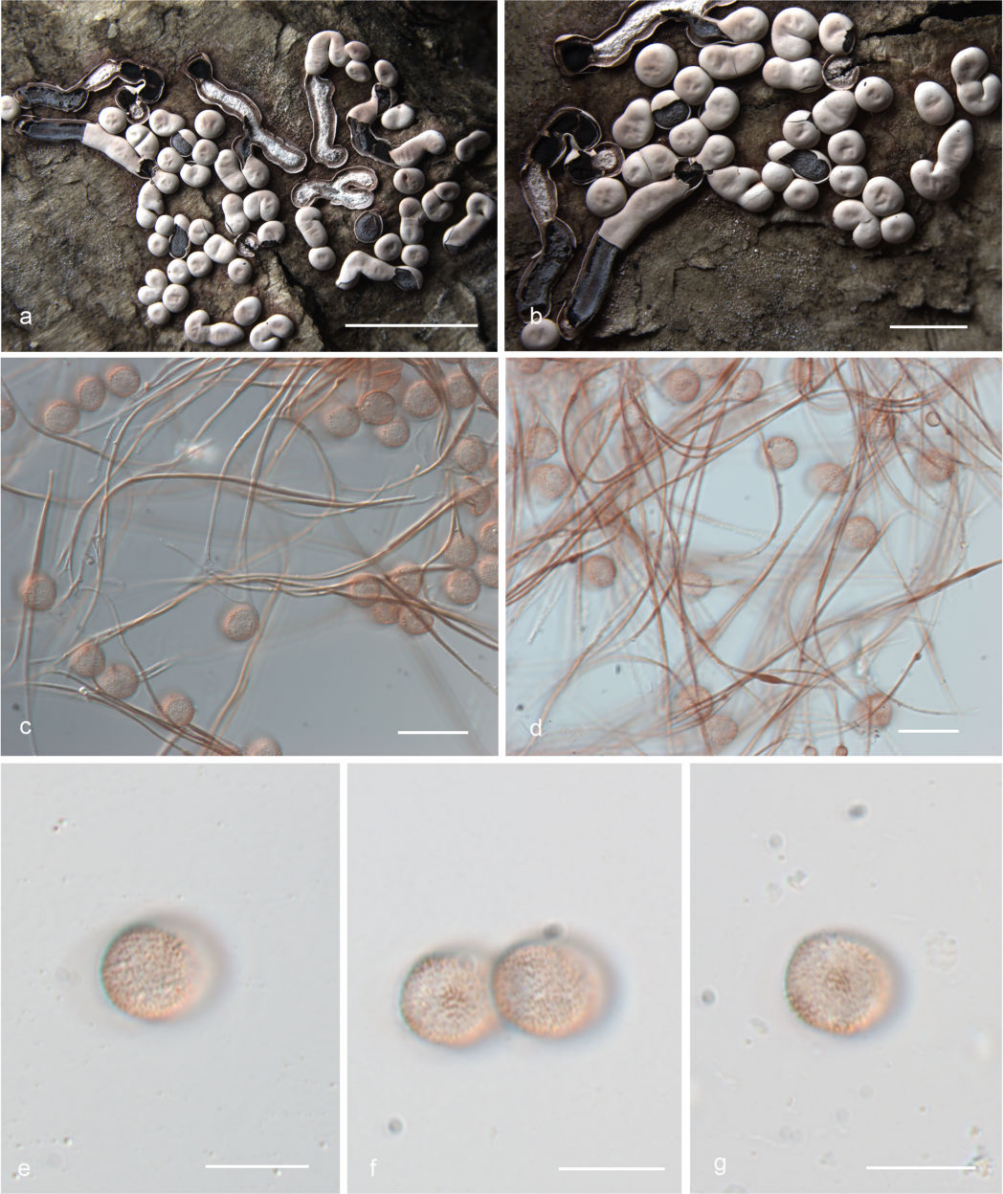
Habitat and microstructure of *Diderma roseum* (HMJAU M20046 Holotype). (a and b) Sporangia to plasmodiocarpous; (c and d) capillitium and some spores by transmitted light (TL); e.g., spores by transmitted light. Scale bars: a = 5 mm; b = 2 mm; c and d = 20 µm; e–g = 10 µm.

***Diderma roseum*** X.F. Li, B. Zhang & Y. Li, sp. nov. (Fig. 3).

MycoBank: MB 852953.

Etymology: the epithet “*roseum*” refers to the rose pink or flesh pink peridium of this species.

Diagnosis: rose pink or flesh pink sporangia, brown and rough with nodules capillitium.

Holotype: China, Shaanxi Province, Baoji City, Meixian County, Taibai Mountain National Forest Park National Forest Park, on the rotten woods, 22 July 2014, Bo Zhang, HMJAU M20046.

Description: sporangia to plasmodiocarpous, sessile, hemispherical, (0.8–) 0.9–1.4 (–1.5) mm, plasmodiocarpous up to 6.4 mm, gregarious or scattered, pink, rose pink, or flesh pink. Peridium is typically double-layered, the outer layer calcareous, fragile, smooth, thick, composed of a tightly packed layer of calcareous lime granules, the inner layer membranous, gray or gray-white, furrowed, sometimes with iridescent, separated from the outer layer. Columella yellow-brown or flesh-colored, calcareous, hemispherical, or pulvinate, forming a thin layer at the base of the sporacarps. Hypothalus inconspicuous. Capillitium is dense, brown, colorless at the end, rough with circular nodules, and branches, and connected, with a membrane enlargement sheet. Spore-mass dark, light brown under transmitted light (TL), (8.5–) 9–10 µm in diameter, with warted.

Distribution in China: Guangdong Province, Jilin Province, Shaanxi Province.

Habitat: on the rotten woods, branches, and leaves.

Additional specimens examined: China, Shaanxi Province, Baoji City, Meixian County, Taibai Mountain National Forest Park, on the rotten branches and leaves, 22 July 2014, Bo Zhang, HMJAU M20047, HMJAU M20052, HMJAU M20048; China, Jilin Province, Yanbian Korean Autonomous Prefecture, Antu County, Changbai Mountain National Nature Reserve, on the rotten branches and leaves, 13 August 2013, Bo Zhang, HMJAU M20044; China, Jilin Province, Tonghua City, Huinan County, Sanjiaolongwan Nature Reserve, on the rotten branches and leaves, 8 August 2022, Xuefei Li, HMJAU M20049; China, Guangdong Province, Shaoguan City, Shixing County, Chebaling National Nature Reserve, on the rotten branches and leaves, 25 June 2013, Bo Zhang, HMJAU M20045.

Notes: the main characteristic of this species is pink or flesh-pink sporangia to plasmodiocarpous, two-layered peridium, hemispherical, or pulvinate columella. *D. testaceum* is similar to *D. roseum* because of its sporocarp color, spore size, and columella color (15). However, some morphological features of *D. roseum* differ from *D. testaceum*, which has small sporangia (0.7–1 mm in diameter), pink, often fading to white, capillitium smooth, light or colorless, and spores nearly smooth (2). In contrast, *D. roseum* has large sporangia (0.9–1.4 mm in diameter), plasmodiocarpous up to 6.4 mm, pink, rose pink, or flesh pink, capillitium with nodules, brown and rough, and spores with obvious warts. In addition, *D. roseum* is similar to *D. carneum*, *D. rufum,* and *D. sauteri*, but *D. roseum* has little spores (9–10 μm), and others have large spores (10–16 μm) (2, 15).

**Fig 4:**
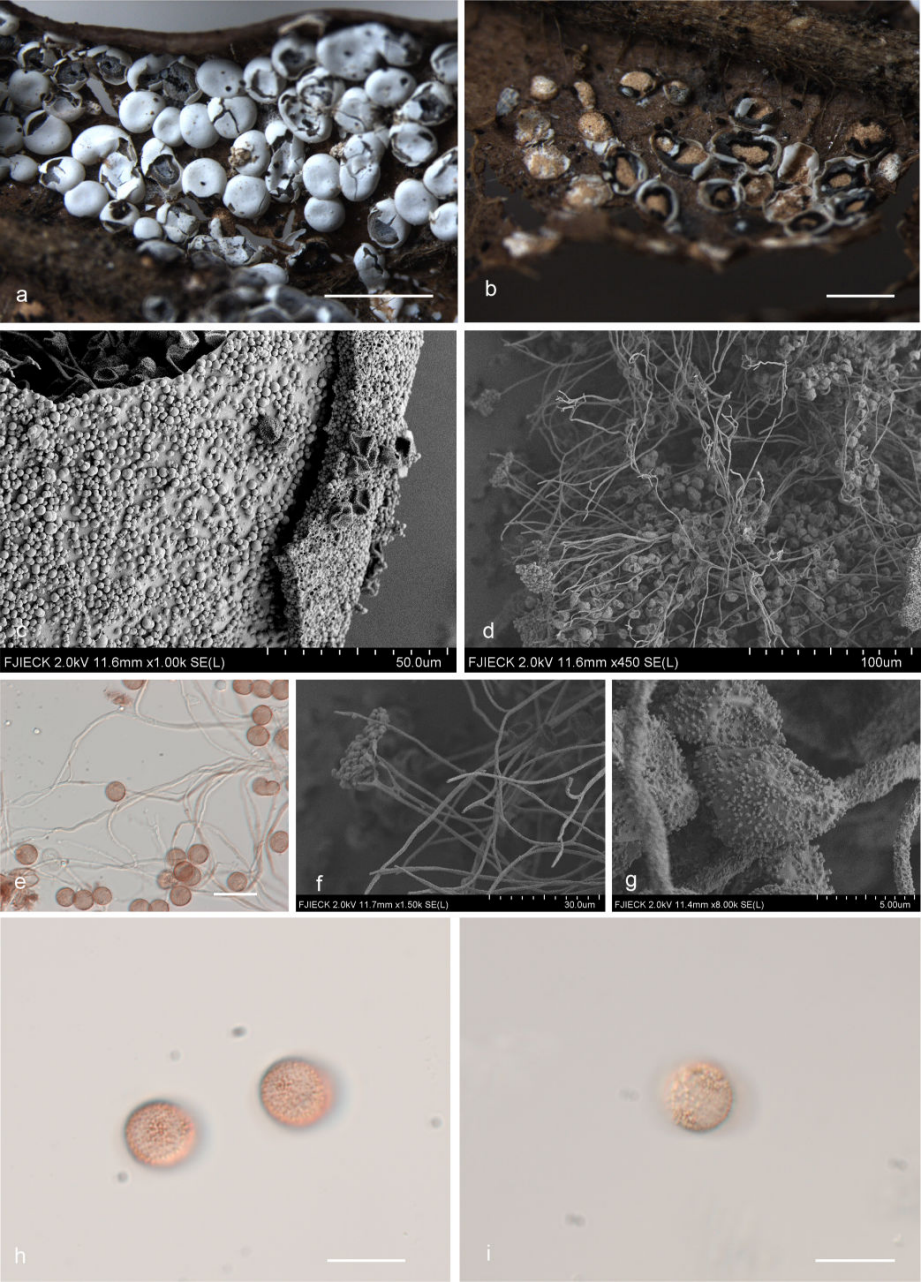
Habitat and microstructure of *D*. *jilinense* (HMJAU M20043 Holotype). (a and b) Sporangia and columella; (c) peridium by scanning electron microscope (SEM); (d–f) capillitium and some spores by TL and SEM; (g–i) spores by TL and SEM. Scale bars: a = 2 mm; b = 1 mm; e = 20 µm; h and i = 10 µm.

***Diderma jilinense*** X.F. Li, B. Zhang & Y. Li, sp. nov. (Fig. 4).

MycoBank: MB 852954.

Etymology: the epithet “*jilinense*” refers to Jilin, the location of the holotype.

Diagnosis: peridium is often broken into irregular fragments.

Holotype: China, Jinlin Province, Changchun City, Jilin Agricultural University campus, on the rotten leaves, 29 June 2022, Xuefei Li, HMJAU M20043.

Description: sporangia, sessile, spherical or ellipsoidal, gregarious, deformation caused by mutual squeezing, white. Peridium is double-layered, the outer layer calcareous, white, smooth, brittle, the inner layer membranous, winkle, gray-white, shining, separated from the outer layer, irregular dehiscent, often broken into fragments. Columella developed and conspicuous, pulvinate, yellowish-brown. Hypothallus scarcely developed, calcareous, white. Capillitium is colorless, dense, and smooth, with an enlarged membrane, branching and connecting. Spore-mass dark, light brown under TL, 8–9 (10) μm in diameter, with warted.

Habitat: on the rotten leaves.

Distribution in China: Jilin Province.

Additional specimens examined: China, Jinlin Province, Changchun City, Jilin Agricultural University campus, on the rotten leaves, 29 June 2022, Xuefei Li, HMJAU M20053, HMJAU M20054; China, Jinlin Province, Changchun City, Jilin Agricultural University campus, on the rotten leaves, 28 June 2022, Xuefei Li, HMJAU M20042; China, Shaanxi Province, Baoji City, Meixian County, Taibai Mountain National Forest Park, on the rotten leaves, 22 July 2014, Bo Zhang, HMJAU M20020, HMJAU M20022; China, Shaanxi Province, Niubeiliang National Nature Reserve, on the rotten leaves, 24 July 2014, Bo Zhang, HMJAU M20021.

Notes: *D. jilinense* can be characterized by its calcareous outer layer and membranous inner layer, irregularly dehiscent, and often broken into fragments, developed and conspicuous columella. Morphologically, *D. jilinense* is related to *D. mussooriense* and *D. alpinum* (2, 3, 15). *D. jilinense* can be distinguished from *D. mussooriense* by its spherical or ellipsoidal sporangia, pulvinate columella, and colorless capillitium (2, 3). *D. jilinense* is similar to *D. alpinum* in its subglobose sporocarpic, two-layered peridium (cartilaginous outer layer and membranous inner layer), and pilvinate columella, but *D. jilinense* differs from *D. alpinum* in a small spore, peridium often broken into fragments, and colorless, smooth capillitium (2, 15).

**Fig 5:**
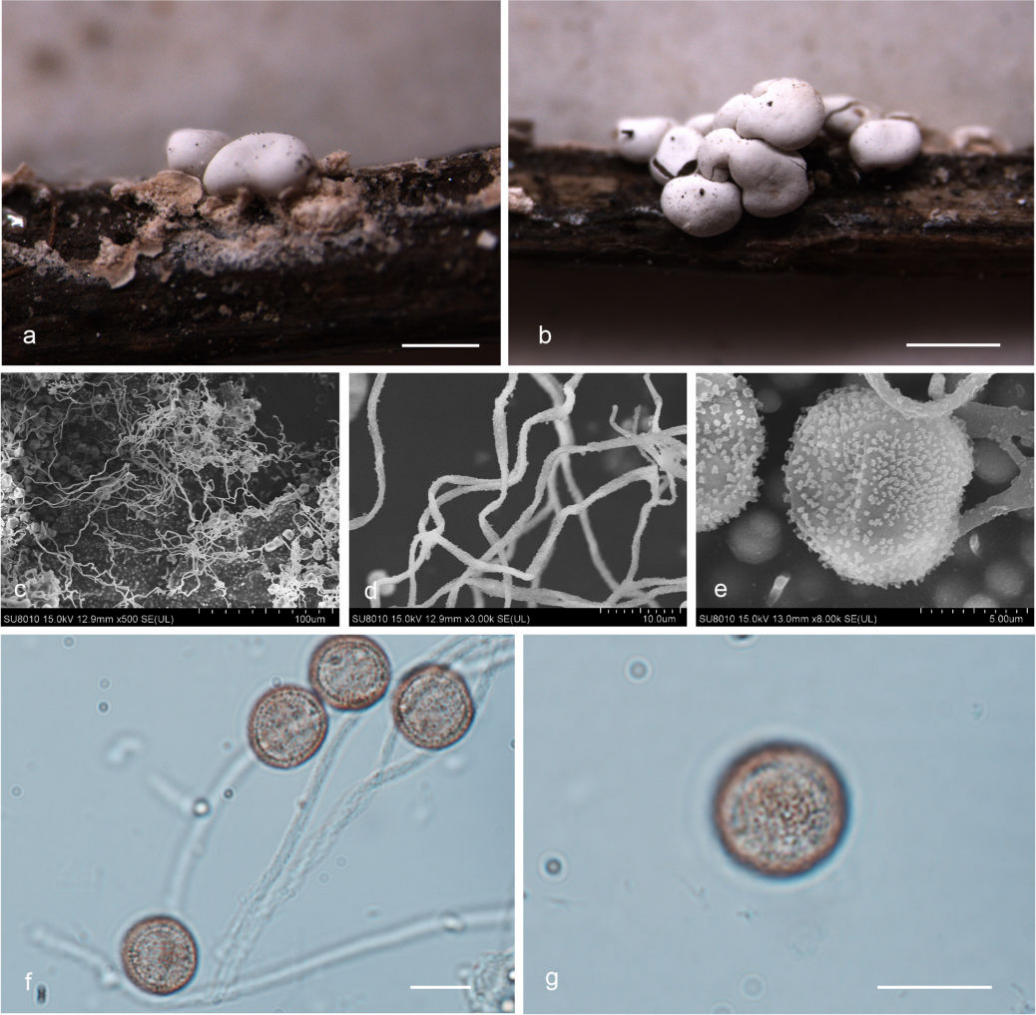
Habitat and microstructure of *D. gansuense* (HMJAU M20013 Holotype). (a) Columella; (b) sporangia; (c–e) capillitium and spores by SEM; (f and g) spores with small and clumps warts by TL. Scale bars: a = 500 µm; b = 1 mm; f and g = 10 µm.

***Diderma gansuense*** X.F. Li, B. Zhang & Y. Li, sp. nov. (Fig. 5).

MycoBank: MB 852956.

Etymology: the epithet “*gansuense*” refers to Gansu, the location of the holotype.

Diagnosis: compared to previously reported species, this species does not have very special characteristics in macroscopical features, but there are some subtle differences, especially in the decoration of spores (small and clumps warts), the color, and decoration of capillitium (rough, with small nodules).

Holotype: China, Gansu Province, Tianshui City, Dangchuan Town forest farm, on the dead leaves and branches, 15 August 2010, Bo Zhang, HMJAU M20013.

Description: sporocarpic scattered to grouped, stacked together, deformed by mutual squeezing, white, smooth, hemispherical or pulvinate, sessile. Peridium is two-layered, the outer layer is limy crust, limy is sparsely scattered on the surface, and the inner layer is tightly attached to the outer layer, membranous. Columella inconspicuous or absent, only a calcareous thickened yellow-brown vesicle base. Capillitium translucent, rough, with small nodules, colorless, fewer branches, bind at the end, about 2 µm in diameter. Spore dark in mass, yellow-brown by transmitted light, with small and clumps warts, 9–11 (–12) µm in diameter.

Habitat: on the dead leaves and branches.

Distribution in China: Gansu Province.

Additional specimens examined: China, Gansu Province, Tianshui City, Dangchuan Town forest farm, on the dead leaves and branches, 19 August 2010, Bo Zhang, HMJAU M21234; China, Gansu Province, Tianshui City, Dangchuan Town forest farm, on the dead leaves and branches, 16 August 2010, Bo Zhang, HMJAU M21235.

Notes: *D. gansuense* is morphologically similar to *D. deplanum* and *D. effusum* due to white and smooth sporocarpic, two-layered peridium, and inconspicuous columella (2, 3, 15). However, *D. gansuense* differs from *D. deplanum* and *D. effusum* by its hemispherical or pulvinate sporocarpic, without plasmodiocarp. In micro-structure, *D. gansuense* differs from *D. deplanum* by its spores with small and clumps warts, capillitium colorless with small nodules, while *D. deplanum* has dark purple capillitium and spores with spiny. *D. gansuense* differs from *D. effusum* by its large spores (9–12 µm), while the latter has smaller spores (6–10 µm). In addition, *D. gansuense* is also similar to *D. saundersii*, but the latter columella is absent, capillitium threads thin, and branched, with few anastomoses (2).

**Fig 6:**
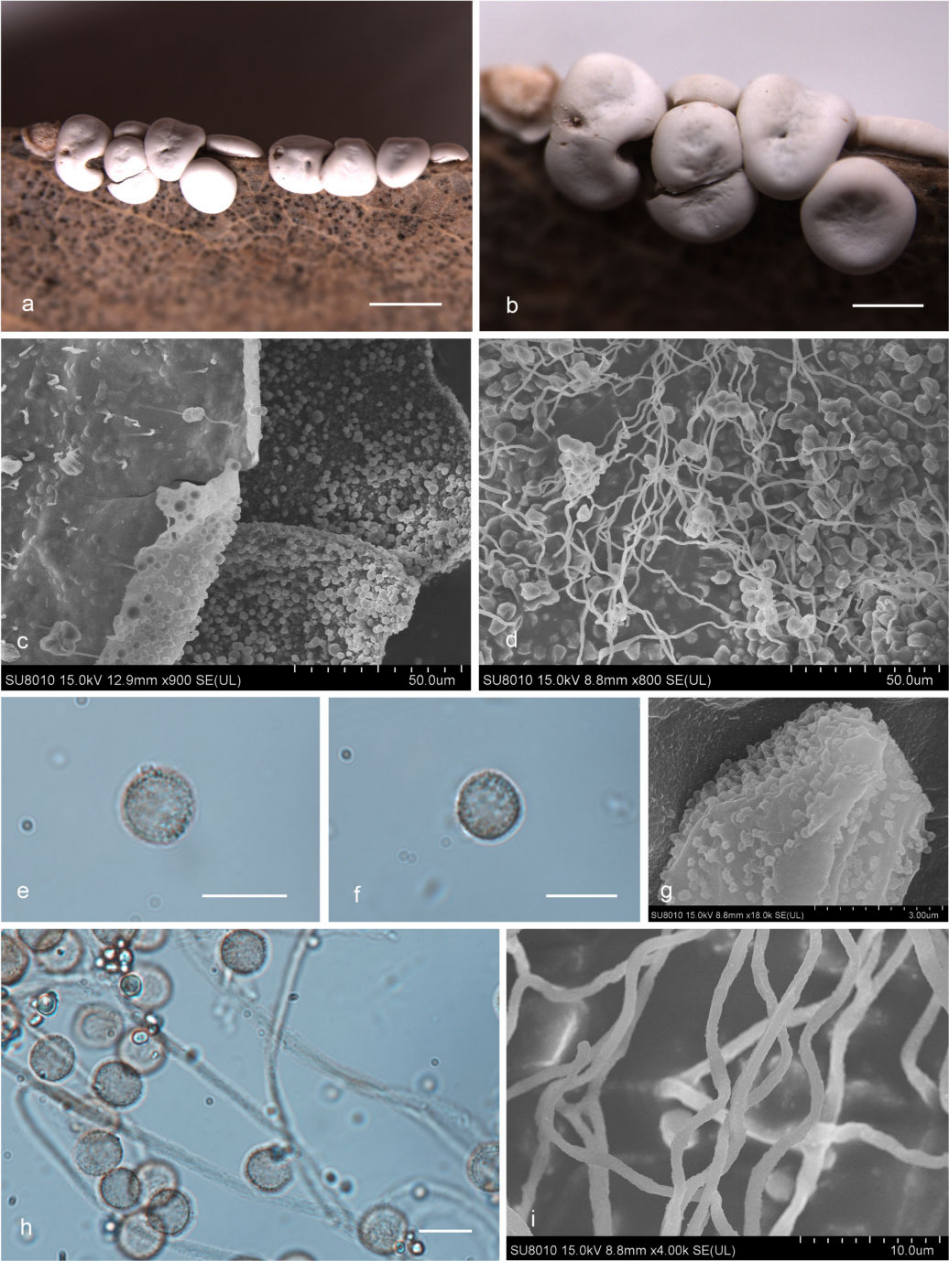
Habitat and microstructure of *D. flexocapillitium* (HMJAU M20102 Holotype). (a and b) Sporangia to plasmodiocarpous; (c) two-layered peridium by SEM; (d) capillitium and some spores by SEM; (e and f) spores by transmitted light; (g) spores marked with warts by SEM; (h) capillitium and some spores by TL; (i) capillitium by SEM. Scale bars: a = 1 mm; b = 500 µm; e, f, and h = 10 µm.

***Diderma flexocapillitium*** X.F. Li, B. Zhang & Y. Li, sp. nov. (Fig. 6).

MycoBank: MB 852957.

Etymology: the epithet “*flexocapillitium*” refers to the soft and flexible capillitium.

Diagnosis: spores with warts and often formed ridge. The capillitium is crude (3 µm), soft, flexible, and twining.

Holotype: China, Jinlin Province, Baishan City, Fusong County, Lushuihe Town Town, on the rotten leaves, 21 July 2012, Bo Zhang, HMJAU M20102.

Description: sporangia to plasmodiocarpous, sessile, pulvinate, (0.5–) 0.6–0.8 (–0.9) mm, plasmodiocarpous up to 1.5 mm. Peridium is typically double-layered, the outer layer calcareous, composed of a tightly packed layer of calcareous lime granules, fragile, smooth, the inner layer membranous, separated from the outer layer. Columella is yellowish brown, roughened, and calcareous, forming a thin layer at the base of the sporacarps. Capillitium crude, with a few branches, soft, and some spiral winding, 3 µm in width, nearly colorless. Spore-mass yellowish-brown, light brown under TL, (7–) 8–9(–10) µm in diameter, with warts and ridge by scanning electron microscope (SEM).

Habitat: on the rotten leaves.

Distribution in China: Jilin Province.

Additional specimens examined: China, Jinlin Province, Baishan City, Fusong County, Lushuihe Town, on the rotten leaves, 21 July 2012, Bo Zhang, HMJAU M20101, HMJAU M20019.

Notes: the primary characteristic of this species is that the capillitium is soft and easily spiral entangled. *D. flexocapillitium* shares similarities with *D. effusum* (Schwein.) Morgan in terms of sporocarp size and capillitium color (2, 15). However, *D. flexocapillitium* can be distinguished from *D. effusum* by its main fomed sporocarpic, with a membranous enlargement sheet, crude capillitium (about 3 µm thick), some spiral winding, an inconspicuous columella, spores with very minutely warted and groups of denser warts, but the later main often extensive plasmodiocarps, thin capillitium (about 1 µm thick), spores with warts and ridge (2, 3).

**Fig 7:**
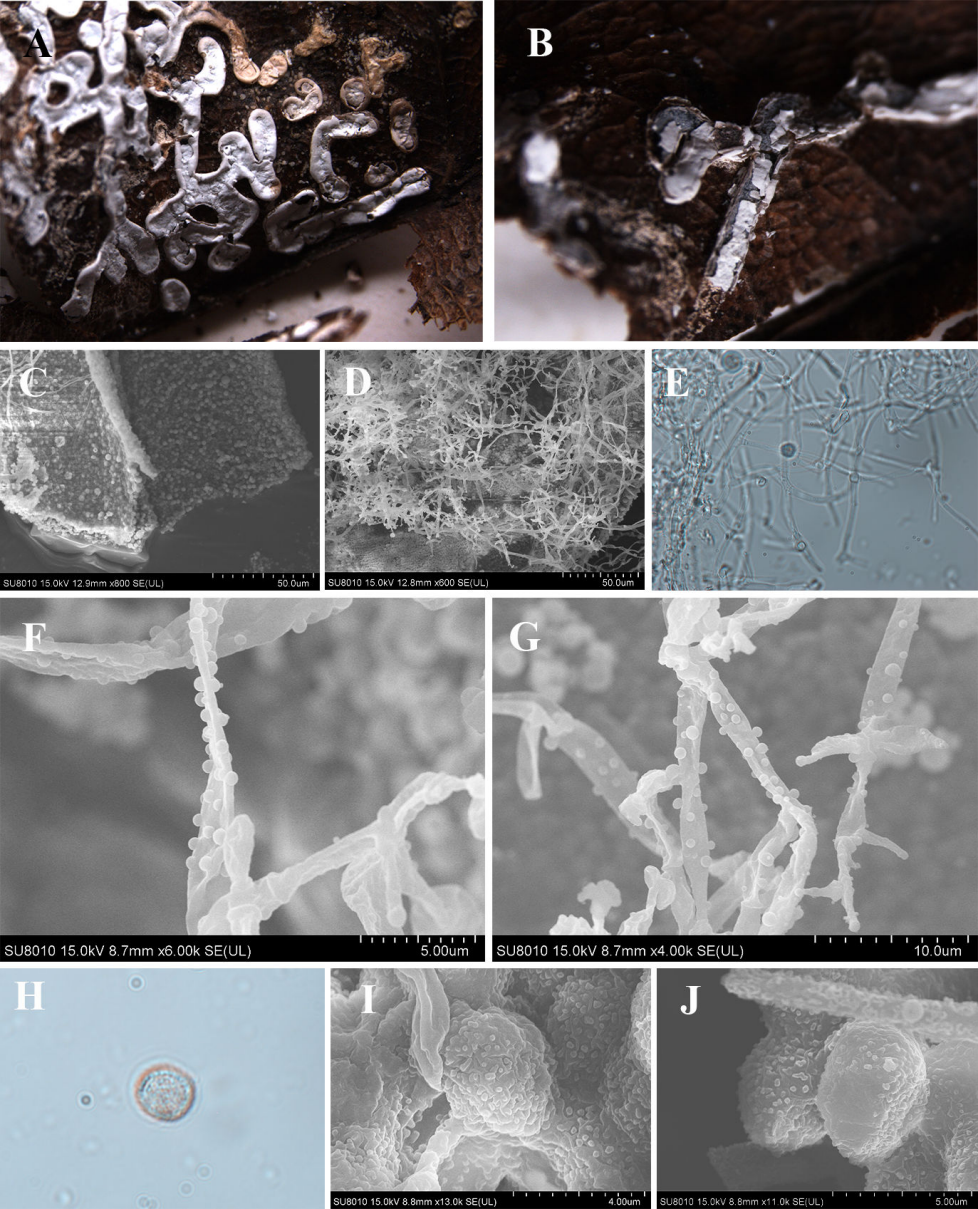
Habitat and microstructure of *D. annuliferum* (HMJAU M20001 Holotype). (A) Plasmodiocarps; (B) columella; (C) two-layered peridium by SEM; (D) capillitium and peridium by SEM; (E) capillitium by TL; (F and G) capillitium with many round ball surrounding by SEM; (H) spore by TL; (I and J) spores by SEM. Scale bars: a = 2 mm; b = 1 mm; e = 20 µm; h = 10 µm.

***Diderma annuliferum*** X.F. Li, B. Zhang & Y. Li, sp. nov. (Fig. 7).

MycoBank: MB 852958.

Etymology: the epithet “*annuliferum*” refers to the capillitium with the ring, by many round balls surrounding it. These spherical patterns are extremely special. As far as we know, no species has such patterns reported so far. Considering the morphological characteristics, it is one of the main features that distinguish other species.

Diagnosis: spores with sparse and big warts. Capillitium with ring.

Holotype: China, Sichuan Province, Ganzi Tibetan Autonomous Prefecture, Gexigou National Reserve, on the rotten branches and leaves, 15 August 2012, Bo Zhang, HMJAU M20001.

Description: sporocarpous to plasmodiocarpous, sessile, pulvinate, 0.5–0.6 (–0.8) mm in diameter, plasmodiocarps extending up to 6 mm in length, 0.5–0.7 (–0.8) mm in width, connected to form a net, edge cracking, and the roof of the plasmodiocarps caved in. Hypothallus white. Peridium is double-layered, the outer layer limy, thick, fragile, edge smooth, the middle part slightly rough and invaginated, the inner layer membranous, transparent, slightly yellow, the two layers closely applied. Columella is yellowish brown, roughened, and calcareous, forming a thin layer covering the base of the sporacarp. Capillitium crude, translucent, tubulose with a ring visible by transmitted light and many round balls surrounding it under a SEM, smooth, 3 µm in wide. Spores not easily dispersed, yellowish-brown in mass, light yellowish brown under a light microscope, (7–) 7.5–9 µm in diameter, with sparse verrucose by SEM.

Habitat: on the rotten branches and leaves.

Distribution in China: Sichuan Province.

Additional specimens examined: China, Sichuan Province, Ganzi Tibetan Autonomous Prefecture, Gexigou National Reserve, on the rotten branches and leaves, 15 August 2012, Bo Zhang, HMJAU M20103, HMJAU M20104.

Notes: *D. annuliferum* is characterized by its light-colored capillitium with a ring or many round balls surrounding it and its spores that are not easily dispersed. Morphologically, *D. annuliferum* shares similarities with *D. effusum*, including sporocarp size and spore diameter (2–6). Both have plasmodiocarps with a diameter of 7–9 µm, the capillitium in both species appears colorless. However, *D. annuliferum* can be distinguished from *D. effusum* by its coarse tubular, ringed, or surrounded by many round balls surrounding the capillitium. In addition, *D. annuliferum* can be distinguished from *D. chonderoderma* by its small spores (7–9 µm), grown on the rotten branches and leaves, double-layered peridium, columella forming a thin layer covering the base of the sporacarp, and spores with sparse verrucose, but layered has large spores (10–15 μm), grown on the moss and lichen, single-layered peridium, columella with stalk and spores with spiny (2–6, 15).

**Fig 8:**
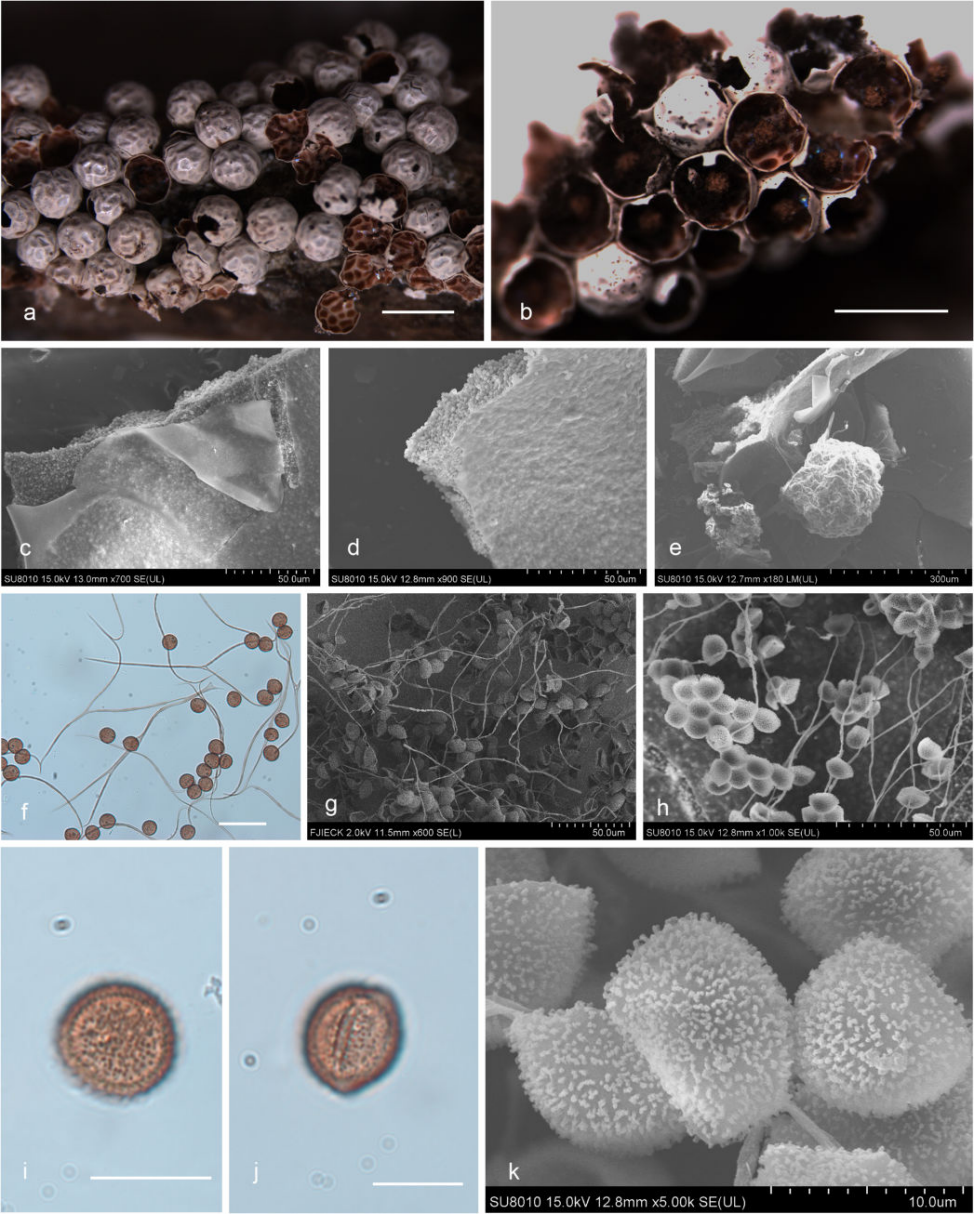
Habitat and microstructure of *D. cor-rubrum* (HMJAU M20010). (a and b) Sporangia; (c and d) two-layered peridium by SEM; (e) columella; (f–h) capillitium and spores by TL and SEM; (i and j) spores with verrucose and ring ridge by TL; (k) spores with warts and ridge by SEM. Scale bars: a and b = 1 mm; f = 40 µm; i and j = 10 µm.

***Diderma cor-rubrum*** T. Macbr., N. Amer. Slime-moulds, ed. 2, 140 (1922) (Fig. 8).

Description: sporocarps, sessile, spherical, (0.6–) 0.7–0.8 (–0.9) mm in diameter, scattered to grouped. Peridium is typically double-layered, the outer layer calcareous, with internal concave, brownish on the inside, fragile, smooth, closely attached to the inner layer, the inner layer membranous, light yellowish brown, shining. Columella brownish, club-shaped, with brown stalk. Capillitium light brown, less, slender, with a membrane enlargement sheet, without swell, branched, and anastomosed. Spores in mass dark brown, brown under light microscope, (10–) 10.5–12 (–13) µm in diameter, with verrucose and ring ridge, like a walnut shell by TL, with warts and ridge by SEM.

Habitat: on the rotten branches and leaves.

Distribution in China: Hebei Province (15), Jilin Province, Liaoning Province, and Yunnan Province (16).

Specimens examined: China, Liaoning Province, Fuxin City, Haitang Mountain Scenic Spot, on the rotten branches and leaves, 1 September 2012, Bo Zhang, HMJAU M20010, HMJAU M20067, HMJAU M20068; China, Jilin Province, Jiaohe City, Lafashan National Forest Park, on the rotten branches and leaves, 6 September 2018, Bo Zhang, HMJAU M20011; China, Liaoning Province, Huludao City, White Wolf Mountain National Nature Reserve, on the rotten branches and leaves, 24 September 2018, Bo Zhang, HMJAU M20012.

Notes: *D. cor-rubrum* is characterized by its brownish inner side of the outer peridium, brownish columella that is club-shaped and stalked, capillitium with a membranous enlargement piece, and spores with annular ridges, resembling a walnut shell in appearance (2, 15). It shares similarities with *D. punense* S. D. Patil, Ranade & R. L. Mishra in some of these characteristics (38, 39). However, some morphological features of *D. cor-rubrum* distinguish it from *D. punense*. Specifically, *D. punense* has large spores ((12.5–) 13–15 µm) with a stalk and a white columella, while *D. cor-rubrum* has small spores ((10.5–) 11-12 (–12.5) µm) with a sessile sporocarp and a tan columella (15, 38, 39).

**Fig 9:**
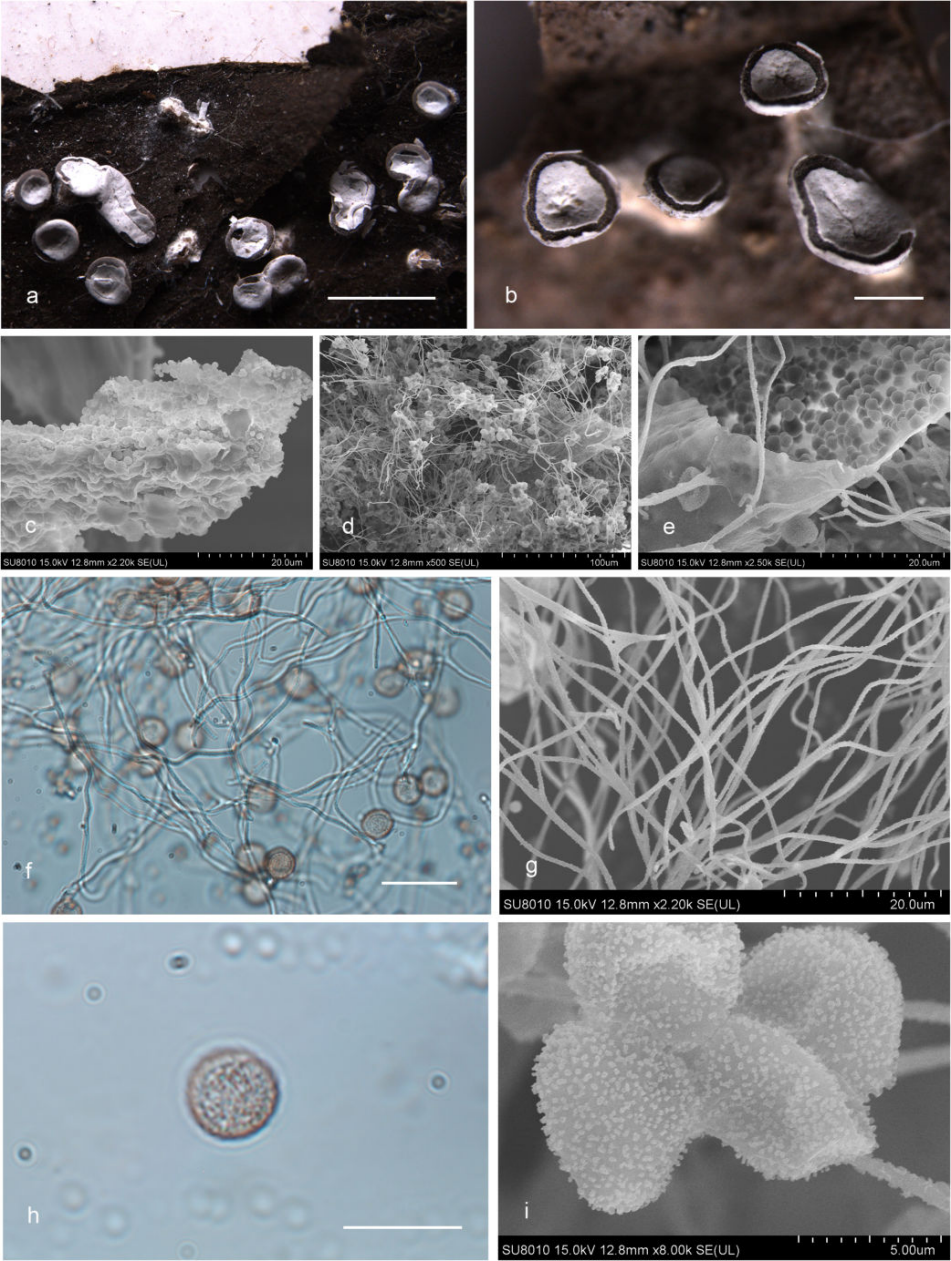
Habitat and microstructure of *D. hemisphaericum* (HMJAU M20017). (a and b) Sporangia; (c) two-layered peridium by SEM; (d) capillitium and spores by SEM; (e) capillitium connected with peridium by SEM; (f) capillitium and spores by TL; (g) capillitium by SEM; (h) spores by TL; (I) spores with warts by SEM. Scale bars: a = 2 mm; b = 200 µm; f = 20 µm; h = 10 µm.

***Diderma hemisphaericum*** (Bull.) Hornem., Fl. dan. 11 (33):13, tab. 1972, 1829 (Fig. 9).

Description: sporophores sporocarpic, white, globose, (0.8–) 1–1.5 (–1.6) mm in diameter, stipitate or partial sessile, 0.8–0.9 × 0.2–0.3 mm, ribbed. Peridium two-layered, the outer layer is calcareous, the edge is easily dehiscent, and the inner layer is membranous, grayish-white, tightly fused with the outer. Columella is inconspicuous, with only a thin layer of yellowish at the base. Capillitium branches and connect, with a membrane enlargement sheet, slender in the end, (1.0–) 1.1–1.5 µm in width. Spores are dark brown in mass, and light brown by transmitted light, with warts, 7–8.5 (–9) µm in diameter.

Habitat: on the rotten woods.

Distribution in China: Anhui Province (16), Beijing City (15), Fujian Province (15), Gansu Province, Guangdong Province (16), Guangxi Zhuang Autonomous Region, Hebei Province (15), Henan Province, Hunan Province (16), Hong Kong Special Administrative Region (16), Jiangsu Province (15), Jilin Province, Liaoning Province, Shandong Province, Sichuan Province, Taiwan Province (16), Xizang Autonomous Region, and Yunnan Province (16).

Specimens examined: China, Gansu Province, Tianshui City, on the rotten leaves, June 2011, Bo Zhang, HMJAU M20017; China, Sichuan Province, Chengdu city, Tazishan Park, on the rotten woods, 15 July 2013, Bo Zhang, HMJAU M20110; China, Henan Province, Zhumadian City, Miyang County, Wanfeng Temple, on the rotten woods, 12 June 2023, Bo Zhang, HMJAU M20111; China, Jilin Province, Tonghua City, Duling Service Area, on the rotten woods, 3 August 2023, Bo Zhang, HMJAU M20112; China, Jilin Province, Changchun City, Jingyuetan National Forest Park, on the rotten woods, 1 August 2023, Bo Zhang, HMJAU M20113; China, Henan Province, Xinyang City, Jigongshan National Nature Reserve, on the rotten woods, 1 July 2015, Bo Zhang, HMJAU M20114; China, Liaoning Province, Fuxin City, Haitangshan National Nature Reserve, on the branches and leaves, 1 September 2012, Bo Zhang, HMJAU M20115; China, Shandong Province, on the leaves, 27 January 1905, Yu Li, HMJAU M21225.

Notes: this species is usually shortly stipitate, but it can also be sessile. It has a wide distribution range. *D. hemisphaericum* is similar to *Didymium clavus* (Alb. & Schwein.) Rabenh. in sporocarp shape, but they differ significantly in calcareous structure (5, 6). *D. clavus* is a crystal, while *D. hemisphaericum* appears as an amorphous particle.

**Fig 10:**
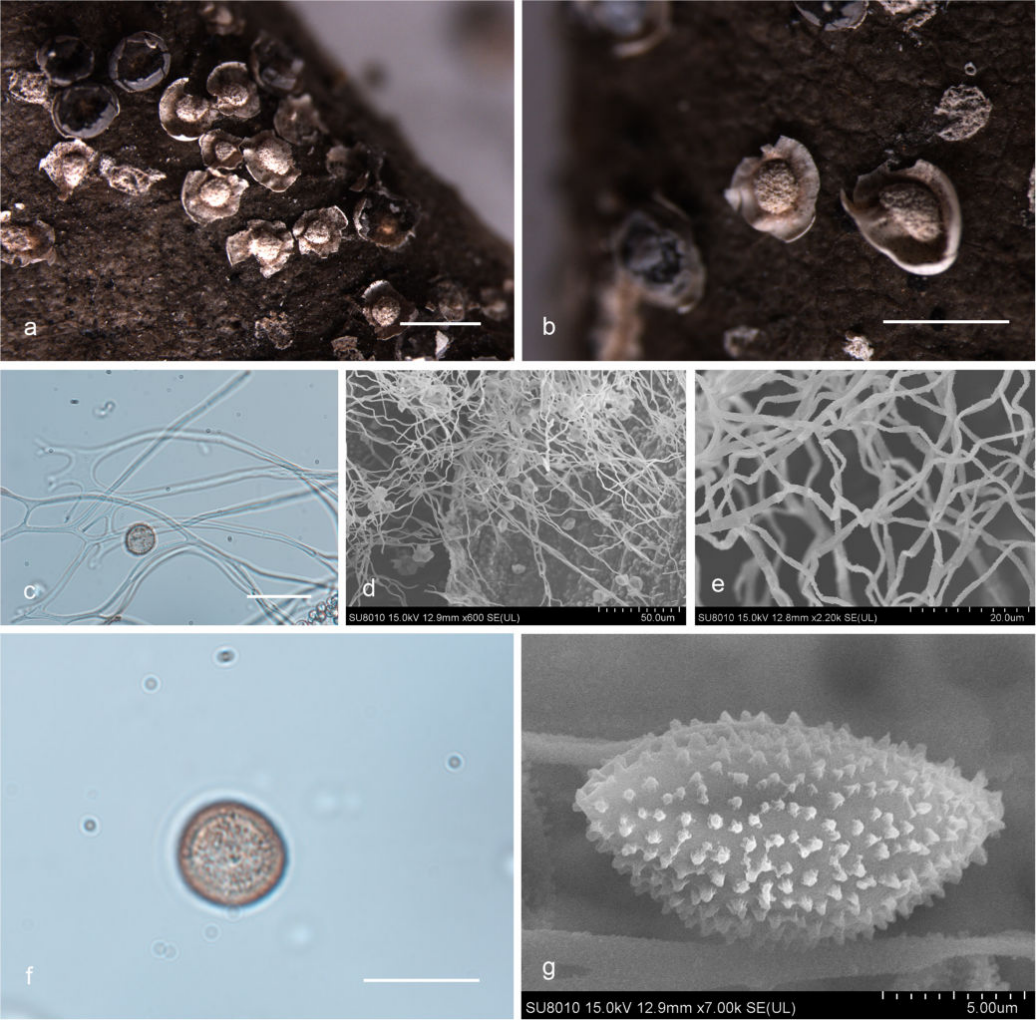
Habitat and microstructure *D. testaceum* (HMJAU M20029). (a and b) Sporangia and columella; (c) capillitium and spores by TL; (d and e) capillitium by SEM; (f and g) spores with warts by TL and SEM. Scale bars: a and b = 1 mm; c = 20 µm; f = 10 µm.

***Diderma testaceum*** (Schrad.) Pers., Syn. meth. fung. 1:167, 1801 (Fig. 10).

Description: sporocarps abundant gregarious, dense, sessile, (0.65–) 0.7–1 (–1.1) mm in diameter, hemispherical, pale pink or white, irregularly dehiscence, often borne on the white hypothallus. Peridium is two-layered, the outer layer is calcareous, smooth, thick, and brittle, and the inner layer is membranous with white lime granules. The columella is large, connected with peridium and hypothallus, hemispherical or extends to the edge, the base at the edge of the columella is pink, iridescent, pale yellow to orange. Capillitium is dense, grayish-white, about 2.2 µm in diameter, few branch connections, and no expansion. Spore (7.5–) 8–9 (–10) µm in diameter, dark brown in mass and brown by TL, warts often clump.

Habitat: on the rotten leaves.

Distribution in China: Anhui Province (16), Gansu Province (16), Henan Province, Hubei Province, Hong Kong Special Administrative Region (16), Jilin Province, Liaoning Province (15), Shanxi Province (15), and Taiwan Province (16).

Specimens examined: China, Henan Province, Sanmenxia City, Lushi County, Shiziping township, on the rotten leaves, 22 September 2013, Bo Zhang, HMJAU M20029; China, Jilin Province, Jiaohe City, Lafashan National Forest Park, on the rotten leaves, 31 July 2022, Xuefei Li, HMJAU M20030; China, Hubei Province, Shiyan City, Fang County, on the rotten leaves, 18 September 2013, Bo Zhang, HMJAU M20031.

Notes: *D. testaceum* was originally described by Schrad (1801) as having a sessile sporocarp, with sporangia undergoing color changes, two-layer peridium with a thick and shelly outer layer, and a membranous inner layer (2, 15). The columella is coarse and densely capillitium. This species has been recorded in several provinces of China (15–17). *D. testaceum* may be confused with *D. difforme* Pers. due to similarities in the shape and size of the sporocarp (6). However, the lime structure of the two species is different. Additionally, attention should be given to the color change of the sporocarp. The Henan and type specimens were found in the same habitat, which consists of dead leaves.

**Fig 11:**
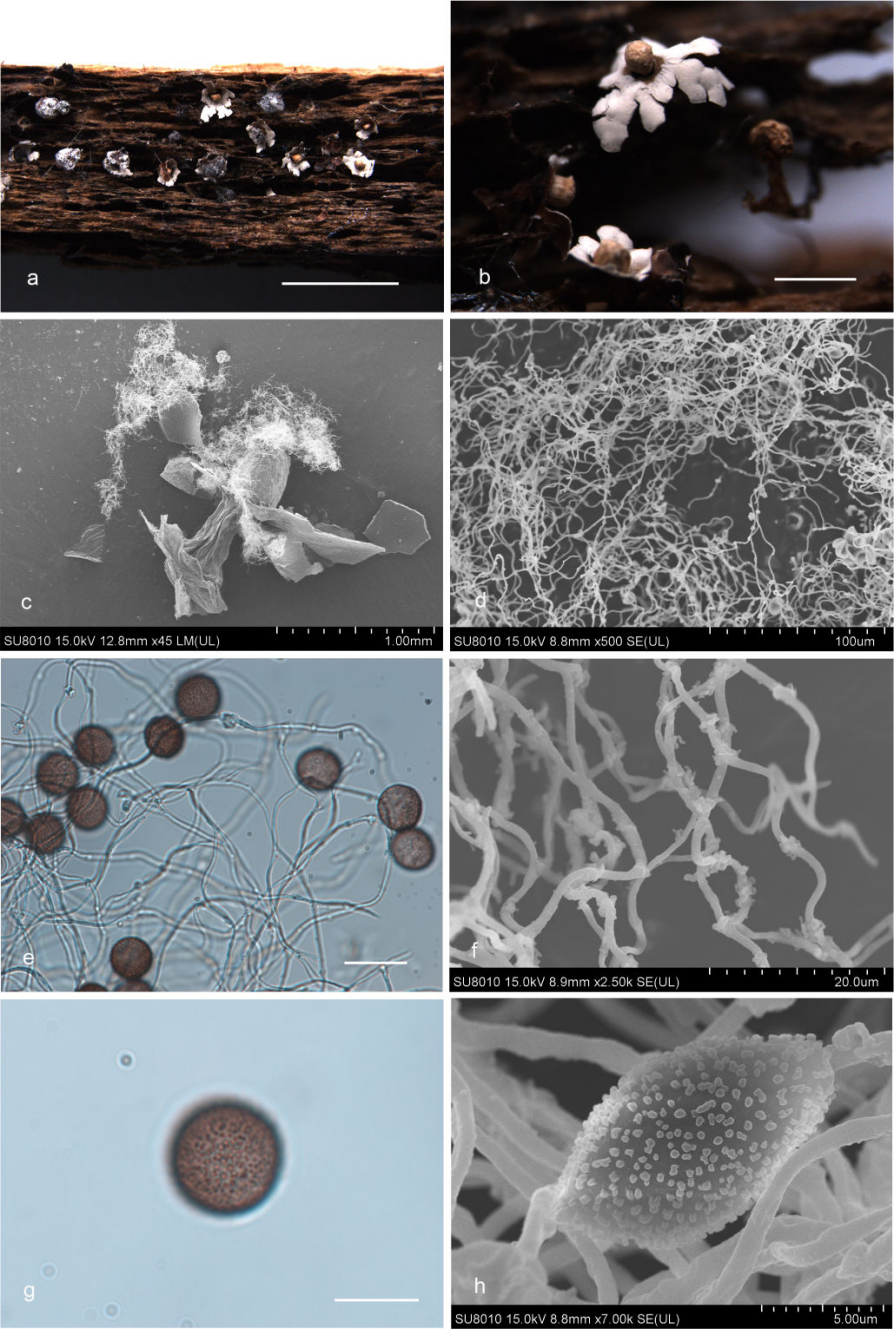
Habitat and microstructure of *D. roanense* (HMJAU M20002). (a and b) Sporangia and columella; (c) sporangia with short stalk by SEM; (d and e) capillitium and spores by SEM and TL; (f) capillitium with swollen knot by SEM; (g and h) spores with warts by TL and SEM. Scale bars: a = 5 mm; b = 1 mm; e = 20 µm; g = 10 µm.

***Diderma roanense*** (Rex) T. Macbr., N. Amer. Slime-moulds, ed. 1, 104, 1899 (Fig. 11).

Description: sporophores sporocarpic, scattered to gregarious, hemispherical to discoid, brown or yellow-brown, with light cracking lines. Sessile or short-stalked, dark, longitudinal groove. Columella present, developed and conspicuous, flat, discoid, ochre-brown, or ivory. Peridium petaloid is dehiscent, two-layered, the outer layer cartilaginous, smooth, the inner layer membranous, white, and tightly fused with the outer layer. Hypothallus inconspicuous. Capillitium sparse, slender, colorless, curved, rarely bifurcated and connected. Spores dark brown in mass, yellow-brown by transmitted light, with obvious warts, (10.5–) 11– (13.5 (–14) µm in diameter.

Habitat: on the rotten woods.

Distribution in China: Hubei Province, Inner Mongolia Autonomous Region, and Qinghai Province (16).

Specimens examined: China, Inner Mongolia Autonomous Region, Hinggan League, Arxan, Motianling, on the rotten woods, 15 August 1985, *Y*u Li & Shuanglin Chen, HMJAU 8877, HMJAU 9130; China, Inner Mongolia Autonomous Region, Hinggan League, Genhe City, 207 Management and Protection Station of Forestry Bureau, on the rotten woods, 4 September 2021, Bo Zhang, HMJAU M20002; China, Inner Mongolia Autonomous Region, Hinggan League, Genhe, on the rotten woods, 5 September 2021, Bo Zhang, HMJAU M20069, HMJAU M20070; China, Hubei Province, Shiyan City, Fang County, on the rotten woods, 18 September 2013, Bo Zhang, HMJAU M20003.

Notes: *D. roanense* can be characterized by its dark short-stalk with a longitudinal groove, cartilaginous outer layer, membranous inner layer, petaloid dehiscence, large and conspicuous columella. Morphologically, *D. roanense* is related to *D. radiatum* and *D. floriforme* (2, 15). *D. roanense* can be distinguished from *D. radiatum* by its short stalk without lime granules, colorless capillitium, and large spores. *D. roanense* is similar to *D. floriforme* by its stalk sporocarpic, dehiscence floriform and two-layered peridium (cartilaginous outer layer and membranous inner layer), but *D. roanense* differs from *D. floriforme* in having a short-stalk, discoid columella, colorless capillitium, and a mottled with brown peridium (2, 15).

**Fig 12:**
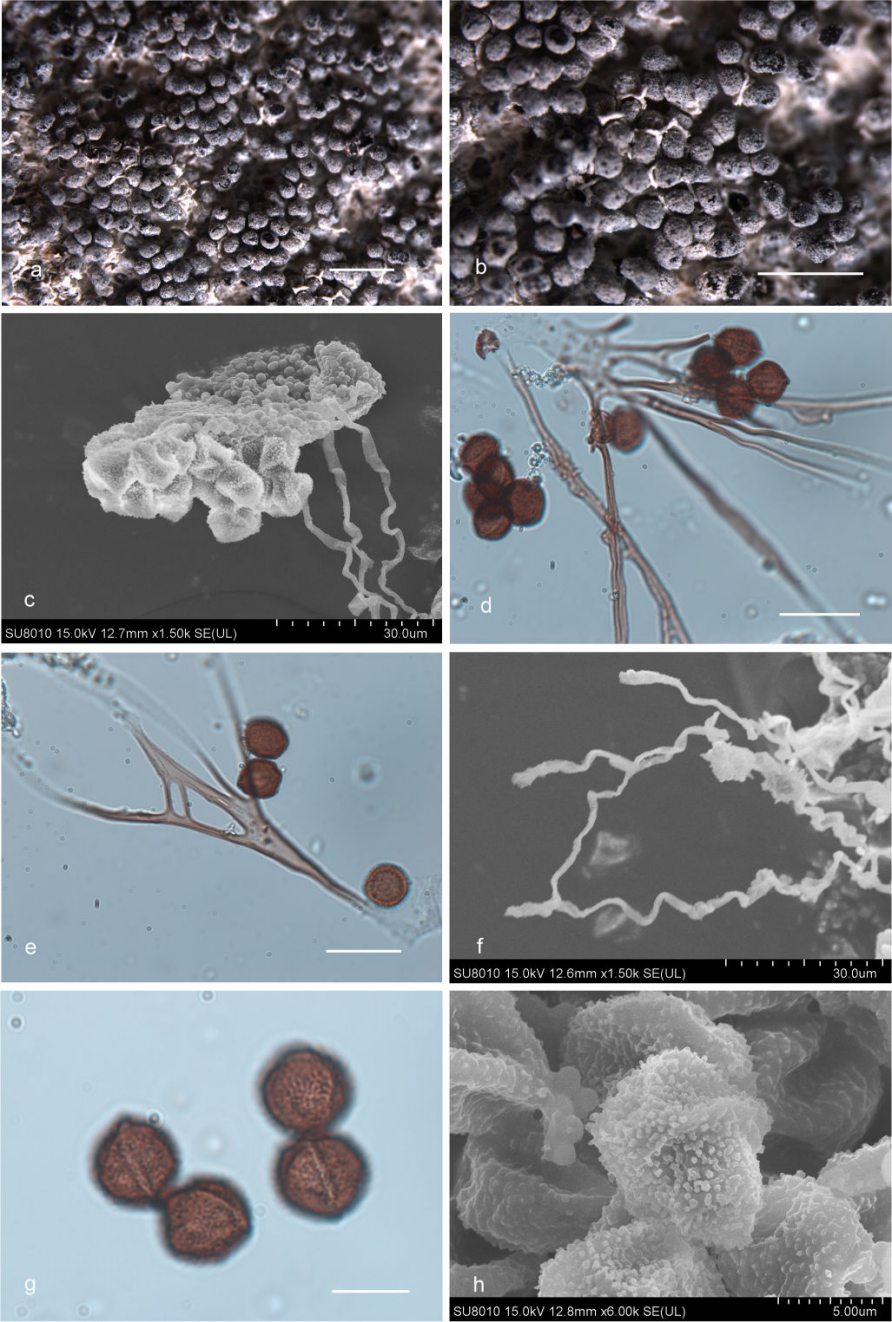
Habitat and microstructure of *D. cingulatum* (HMJAU M20004). (a and b) Sporangia; (c) peridium connected with capillitium; (d and e) capillitium and spores by TL; (f) capillitium by SEM; (g and h) spores with warts and ridge by TL and SEM. Scale bars: a and b = 1 mm; d and e = 20 µm; g = 10 µm.

***Diderma cingulatum*** Nann.-Bremek., Proc. Kon. Ned. Akad. Wetensch., C. 71 (2):191, 1968 (Fig. 12).

Description: sporophores sporocarpic, sessile, subglobose or oblate, gregarious. Hypothallus calcareous, whitish, connected with the base of the peridium and columella. After the sporocarp falls off, the vestige can be seen. Peridium two-layered, the outer layer calcareous, with lime granules, easy to irregular dehiscence and fall off, the inner layer membranous incompletely separate from the outer layer, covered with a layer of white limy granules. Columella present or absent, reaching to the top of the sporocarp when it exists, thin cylindrical, or slightly flat, white. Capillitium brown, uneven in thickness, (2.1–) 2.2–2. 6 (–2.8) µm in width, wavy and curved, and one end is connected with the peridium, like a bunch of flowers by transmitted light. Two ends of the capillitium are light in color, transparent, rarely connected, and have brown nodules. Spores are dark brown in mass, reddish brown by transmitted light, globose or polygon, (10–) 11–12.5 (–13) µm in diameter, reticulation and warts on the surface, and a circular and white dehiscence line, or ridge in the middle.

Habitat: on the rotten woods, branches, and leaves.

Distribution in China: Jilin Province.

Specimens examined: China, Jilin Province, Changchun City, Jilin Agricultural University, on the rotten woods, 5 August 2021, Bo Zhang, HMJAU M20004, HMJAU M20071; China, Jilin Province, Changchun City, Jilin Agricultural University, on the dead branches, 7 September 2015, Bo Zhang, HMJAU M20005; China, Jilin Province, Changchun City, Jilin Agricultural University, on the dead leaves and branches, 27 August 2021, Bo Zhang, HMJAU M20006; China, Jilin Province, Changchun City, Jingyuetan National Forest Park, on the dead leaves, 1 September 2015, Bo Zhang, HMJAU M20007.

Notes: *D. cingulatum* is characterized by sporophores with what appears to be two closely appressed layers of peridium, irregular dehiscence, a circular dehiscence line, or a ridge in the middle of the spores. Sporophores are sessile, with a calcareous hypothallus that reaches the top of the sporocarp when a columella exists. Additionally, spores with a white equatorial band are distinctive. Other characteristics also differ significantly from other species, making it easy to identify.

**Fig 13:**
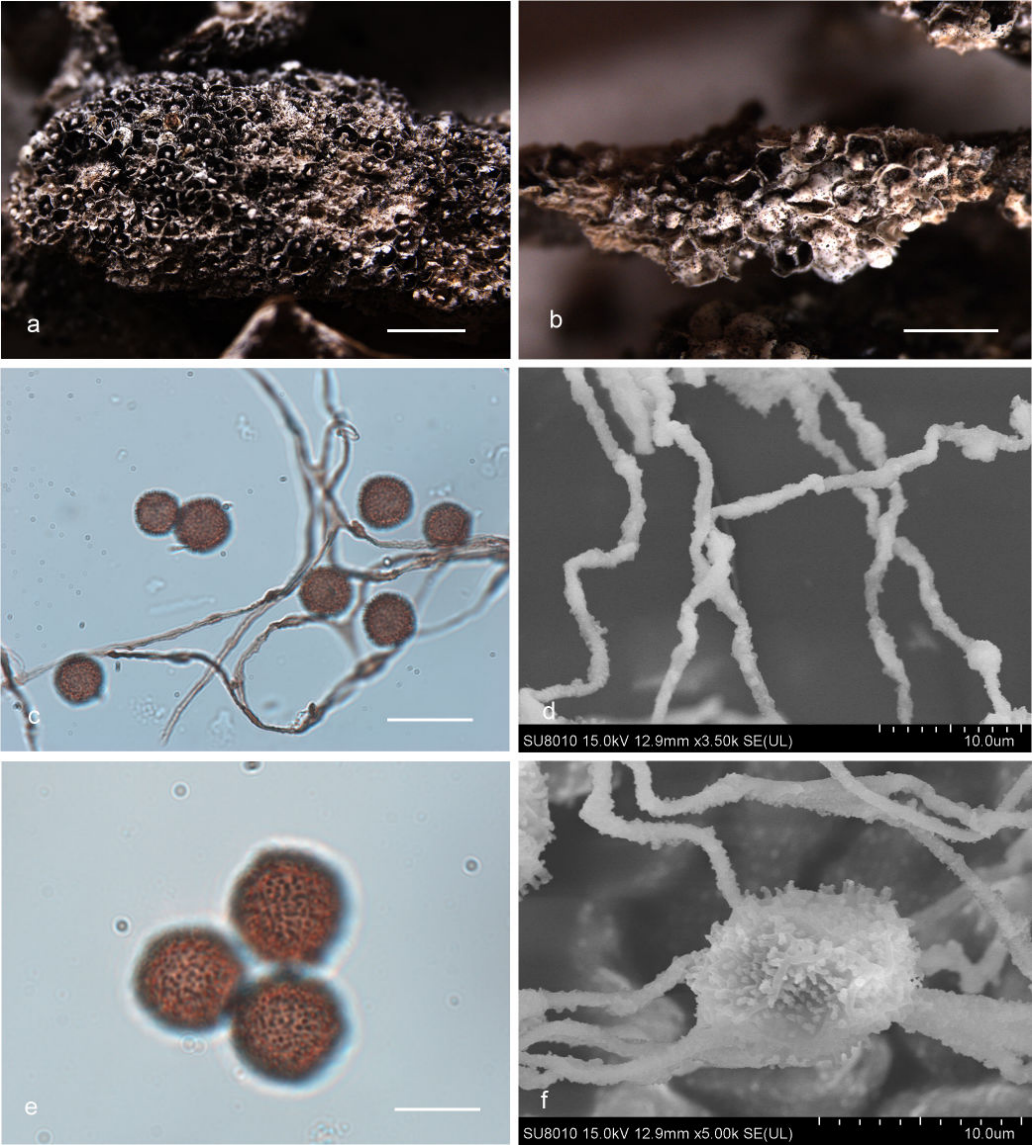
Habitat and microstructure of *D. crustaceum* (HMJAU M20066). (a and b) Sporangia and columella; (c) capillitium and spores by TL; (d) capillitium by SEM; (e and f) spores with warts by TL and SEM. Scale bars: a = 2 mm, b = 1 mm, c = 20 µm, e = 10 µm.

***Diderma crustaceum*** Peck, Bull. Buffalo Soc. Nat. Sci. 1:63, 1873 (Fig. 13).

Description: sporophores sporocarpic, sessile, subglobose or oblate, gregarious, gray-white to white, roof dehiscence. Peridium is two-layered, the outer layer is calcareous, thick, whitish, smooth, and easy to shatter, and the inner layer is membranous, blue with iridescent. Columella present, white or pale yellow, with lime, clavate, hollow. Capillitium darkness, branching and connecting, uneven thickness, (0.6–) 0.7–1 (–1.5) µm in width, swelling up to 3.5 µm. Spores dark gray in mass, yellow-brown by TL, 11–14 (–15) µm in diameter, with warts.

Habitat: on the rotten woods and leaves.

Distribution in China: Beijing City (15), Guizhou Province (16, 18), Hebei Province (15), Heilongjiang Province (15), Inner Mongolia Autonomous Region (15), Jilin Province, and Shanxi Province (16).

Specimens examined: China, Jilin Province, Changchun City, Jilin Agricultural University, on the rotten woods, 13 October 2012, Bo Zhang, HMJAU M20008, HMJAU M20072, HMJAU M20066, HMJAU M20073, HMJAU M20074, HMJAU M20075, HMJAU M20076, HMJAU M20077; China, Jilin Province, Changchun, Jilin Agricultural University, on the rotten leaves, 7 September 2015, Bo Zhang, HMJAU M20009.

Notes: morphologically, *D. crustaceum* resembles *D. subviridifuscum* Buyck and *D. globosum* in its white sporocarps, white hypothallus, and two-layered peridium (40). However, *D. crustaceum* is distinguishable from *D. subviridifuscum* by the color of its inner layer peridium, which is blue with an iridescent sheen, and its clavate shape columella, whereas *D. subviridifuscum* has a greenish-brown and a globose or prolate (40). In terms of microfeatures, the size of the spores is similar among these species, but the capillitium of *D. crustaceum* is dark with flat threads and some swellings. In contrast, the capillitium of *D. subviridifuscum* is pale brown, sometimes enclosing smooth lime globules or having small dark fusiform swellings (40). *D. crustaceum* also differs from *D. globosum* due to its well-developed hypothallus, a clavate columella without a stalk, and large spores (15).

**Fig 14:**
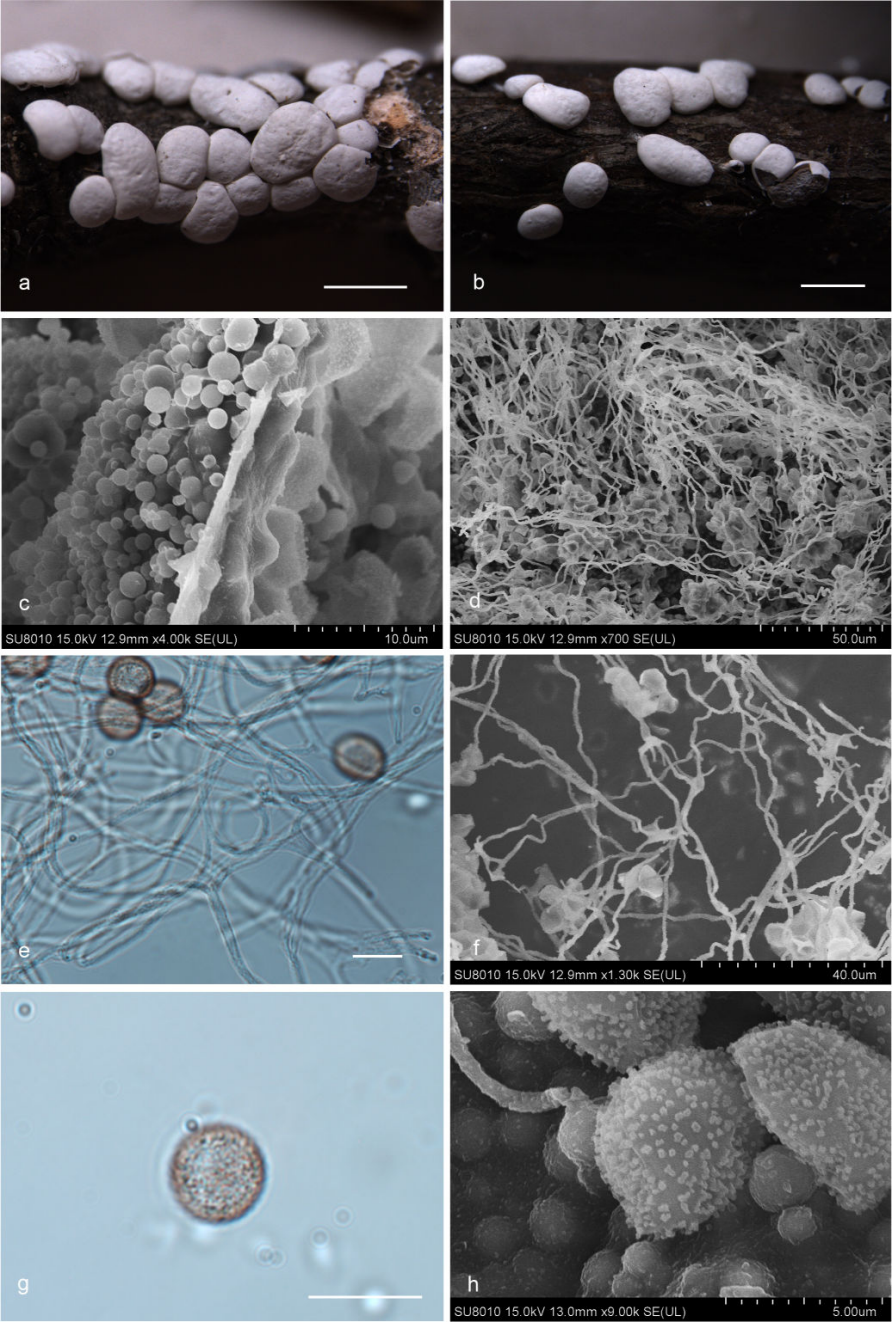
Habitat and microstructure of *D. effusum* (HMJAU M20063). (a and b) Sporangia and columella; (c) two-layered peridium by SEM; (d and e) capillitium and spores by SEM and TL; (f) capillitium by SEM; (g and h) spores with warts by TL and SEM. Scale bars: a and b = 1 mm; e and g = 10 µm.

***Diderma effusum*** (Schwein.) Morgan, J. Cincinnati Soc. Nat. Hist. 16 (4):155, 1894 (Fig. 14).

Description: sporophores sporocarpic to plasmodiocarp, pulvinate, (0.7–) 0.8–2 × 0.5–1.4 (–1.5) mm, whitish. Peridium is two-layered, the outer layer is calcareous, thin, and wrinkled, and the inner layer is membranous, gray, ridged on the surface, easily separate from the outer layer. Columella inconspicuous, pulvinate flattened, a layer of yellow protuberance on the sporocarpic base. Capillitium nearly colorless, (1.3–) 1.5–1.8 (–1.9) µm in width, swollen to (1.7–)1.8–2.1 (–2.2) µm, branching and connecting, thick tubular reticulation on the end. Spores taupe in mass, pinkish brown by transmitted, (6–) 7–8 (–9) µm in diameter, with warts.

Habitat: on the dead leaves and branches.

Distribution in China: Anhui Province (16), Beijing City (15), Fujian Province (15), Gansu Province, Guangdong Province (16), Guangxi Zhuang Autonomous Region (15), Hebei Province (15), Heilongjiang Province (16), Henan Province (16, 17), Hunan Province (16), Hong Kong Special Administrative Region (15), Jiangsu Province (16), Jilin Province, Liaoning Province, Shandong Province (15), Sichuan Province, Taiwan Province (16, 41), Yunnan Province (16), and Zhejiang Province (15).

Specimens examined: China, Jilin Province, Baishan City, Fusong County, Lushuihe Town National Forest Park Hunting Ground, on the dead leaves and branches, 22 July 2012, Bo Zhang, HMJAU M20014, HMJAU M20078, HMJAU M20079, HMJAU M20080, HMJAU M20063, HMJAU M20081; China, Jilin Province, Yanbian Chaoxian Nationality Autonomous Prefecture, Antu County, Erdaobaihe Town, Changbai Mountain National Nature Reserve, on the dead leaves and branches, 5 August 2011, Bo Zhang, HMJAU M20082; China, Jilin Province, Yanbian Chaoxian Nationality Autonomous Prefecture, Antu County, Changbai Mountain National Nature Reserve, Erdaobaihe Town, on the dead leaves, 22 July 2018, Bo Zhang, HMJAU M20015; China, Gansu Province, Tianshui City, Dangchuan Town forest farm, Maiji District, on the dead leaves and branches, 16 August 2010, Bo Zhang, HMJAU M20083, HMJAU M20084; China, Sichuan Province, Ganzi Tibetan Autonomous Prefecture, Yajiang County, Gexigou Nature Reserve, on the dead leaves and branches, 15 August 2012, Bo Zhang, HMJAU M20085; China, Liaoning Province, Fushun City, Qingyuan Manchu Autonomous County, Dasuhe Township, on the dead leaves, 5 September 2012, Bo Zhang, HMJAU M20080.

Notes: morphologically, *D. effusum* shows features of plasmodiocarps, a two-layered peridium, smooth capillitium threads, and spore size that of similar to *D. saundersii* (Berk. & Broome ex Massee) E. Sheld (2–6). However, *D. effusum* can be distinguished from *D. saundersii* by its thicker plasmodiocarps and pulvinate flattened columella, while *D. saundersii* has thin plasmodiocarps and an absent columella (2–6).

**Fig 15:**
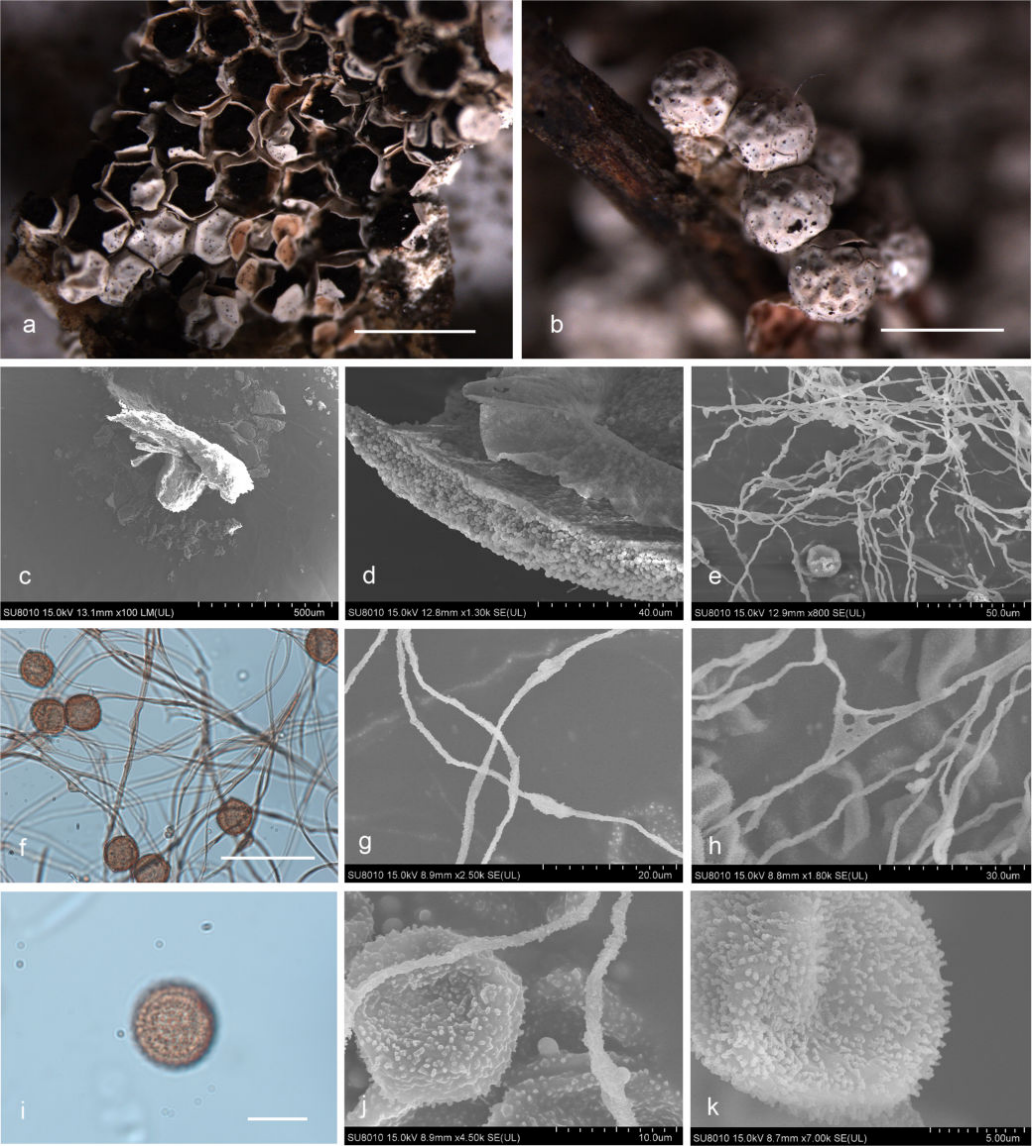
Habitat and microstructure of *D. liaoningensis* (HMJAU M20018). (a-c) Sporocysts and light yellow columella; (d) outer and inner layer of peridium by TL and SEM; (e and f) capillitium and some spores by SEM and TL; (g and h) capillitium by SEM; (i-k) spores with warts by TL and SEM. Scale bars: a = 2 mm; b = 1 mm; f = 40 µm; i = 10 µm.

***Diderma liaoningensis*** H. N. Zhao, B. Zhang, and Yu. Li, in Zhao, Rao, Yang, Li, Yu, Zhang & Li, Phytotaxa 572 (1):66, 2022 (Fig. 15).

Description: sporophores sporocarpic, globose, (0.7–) 0.8–1 (–1.1) mm in diameter, crowded, yellow-white, sessile. Columella present, clavate, calcareous, pale tawny, uneven surface. Peridium is two-layered, the outer layer calcareous with a divisural line, the inner layer membranous, often tightly fused with the outer layer. Dehiscence petaloid. Capillitium brown, branched, and anastomosed to form a net, fan-shaped expansion to flaky, with obviously swelling and knots. Spores black in mass, brown by transmitted light, globose, (12.5–) 13–14.5 (–15) µm in diameter, with warts by TL and SEM.

Habitat: on decaying branches.

Distribution in China: Liaoning Province.

Specimens examined: China, Liaoning Province, Fuxin City, Haitang Mountain Science Spot, on decaying branches, 2 September 2012, Bo Zhang, HMJAU M20086; China, Liaoning Province, Fuxin city, Haitang Mountain Scenic Spot, on decaying branches, 1 September 2012, Bo Zhang, HMJAU M20087, HMJAU M20088, HMJAU M20089, HMJAU M20090, HMJAU M20091, HMJAU M20092, HMJAU M20065, HMJAU M20093, HMJAU M20094, HMJAU M20018.

Notes: *D. liaoningensis* shares morphological similarities with *D. crustaceum* and *D. cingulatum* in terms of sessile and sporocarpic, globose appearance growing on a white or ivory hypothallus, two-layered peridium, clavate columella and spores with warts. However, it can be differentiated by its larger spore size compared to *D. crustaceum*. Furthermore, *D. liaoningensis* differs from *D. cingulatum* in having globose spores, while *D. cingulatum* spores exhibit a polygonal shape with reticulations on the surface. This species has a relatively limited distribution, being found only in the Liaoning Province of China, and was newly species reported in 2022 (42). Additional SEM observations were conducted to supplement its morphology.

**Fig 16:**
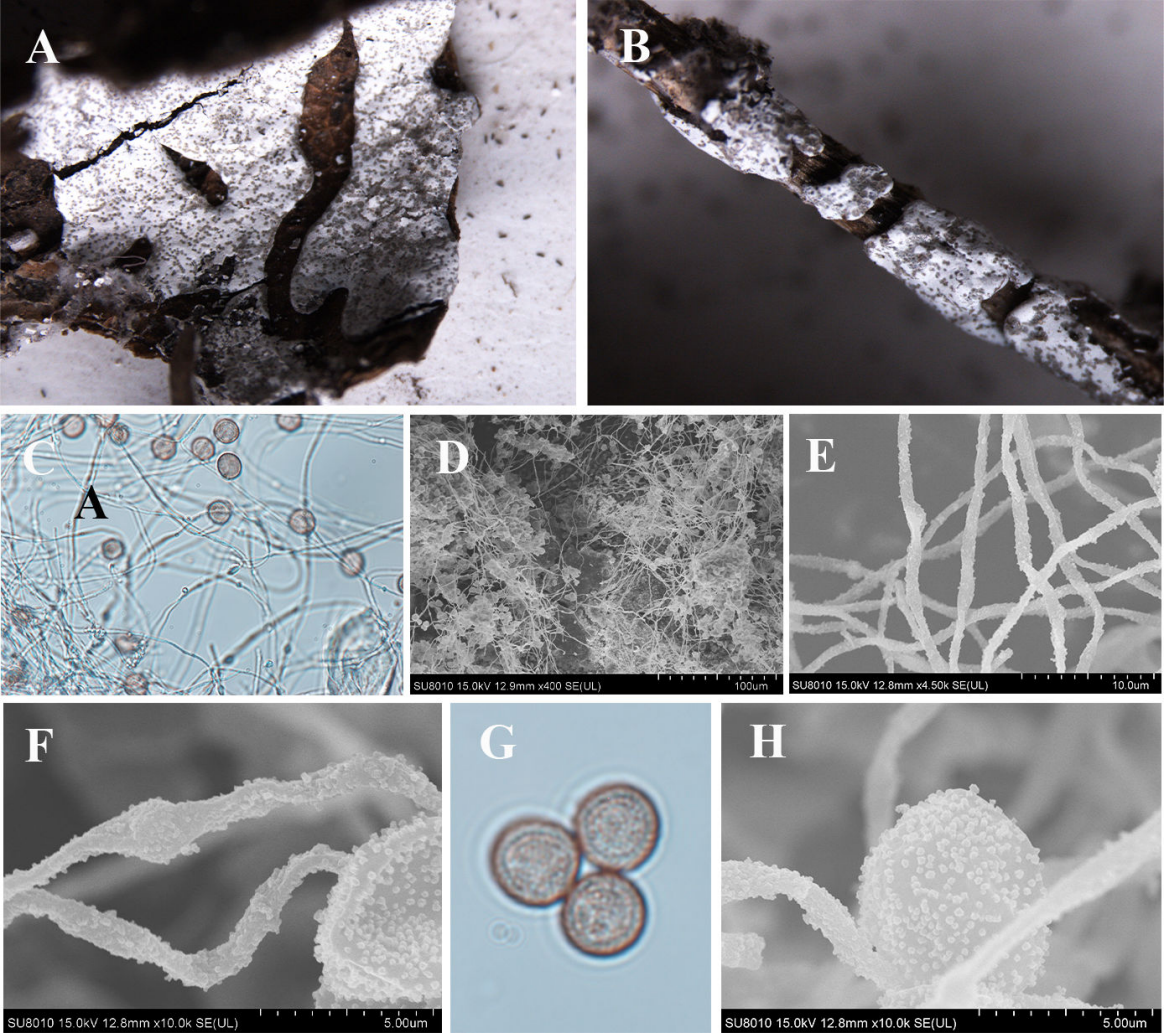
Habitat and microstructure of *D. saundersii* (HMJAU M20027). (A) Sporangia to plasmodiocarpous; (B) columella; (C) capillitium and spores by TL; (D–F) capillitium with swellings and nodules by SEM; (G and H) spores with warts by TL and SEM. Scale bars: a = 2 mm; b = 1 mm; c = 20 µm; g = 10 µm.

***Diderma saundersii*** (Berk. & Broome ex Massee) Ladó, Cuad. Trab. Fl. Micol. Ibérica 16: 35, 2001 (Fig. 16).

Description: sporophores plasmodiocarp, or flat cushion sporocarpic, (0.3–) 0.4–4 (–4.5) × 0.3–5 (–5.2) mm in diameter, grayish white. Hypothallus inconspicuous. Peridium is two-layered, the outer layer is calcareous, sparsely distributed with transparent substances on the surface (calcareous crystals), the inner layer attached to the outer layer but separated, membranous. Columella absent. Capillitium is dense, white, branching and connecting, (1.2–) 1.5–2.5(–2.6) µm in wide, swellings and nodules on it. Spores dark brown in mass, light red gray by transmitted light, 7–8 (–9) µm in diameter, with warts.

Habitat: on the dead branches and leaves.

Distribution in China: Guangdong Province (16), Henan Province (16, 17), Hunan Province (16), Inner Mongolia Autonomous Region (16), Jilin Province, and Taiwan Province (16).

Specimens examined: China, Jilin Province, Changchun City, Jingyuetan National Scenic Area, on the dead branches and leaves, 8 August 2021, Bo Zhang, HMJAU M20027, HMJAU M20116, HMJAU M20117.

Notes: this species is widely distributed, sporocarpic with pulvinate to plasmodiocarp, two-layered peridium, the absence of columella, and colorless capillitium that swells as its main characteristics. *D. saundersii* shares similarities with *D. effusum*, however, it can be distinguished by its pulvinate flattened columella and the thicker plasmodiocarps that often arise from merged sporocarps, distinguishing it from *D. saundersii* (15).

**Fig 17:**
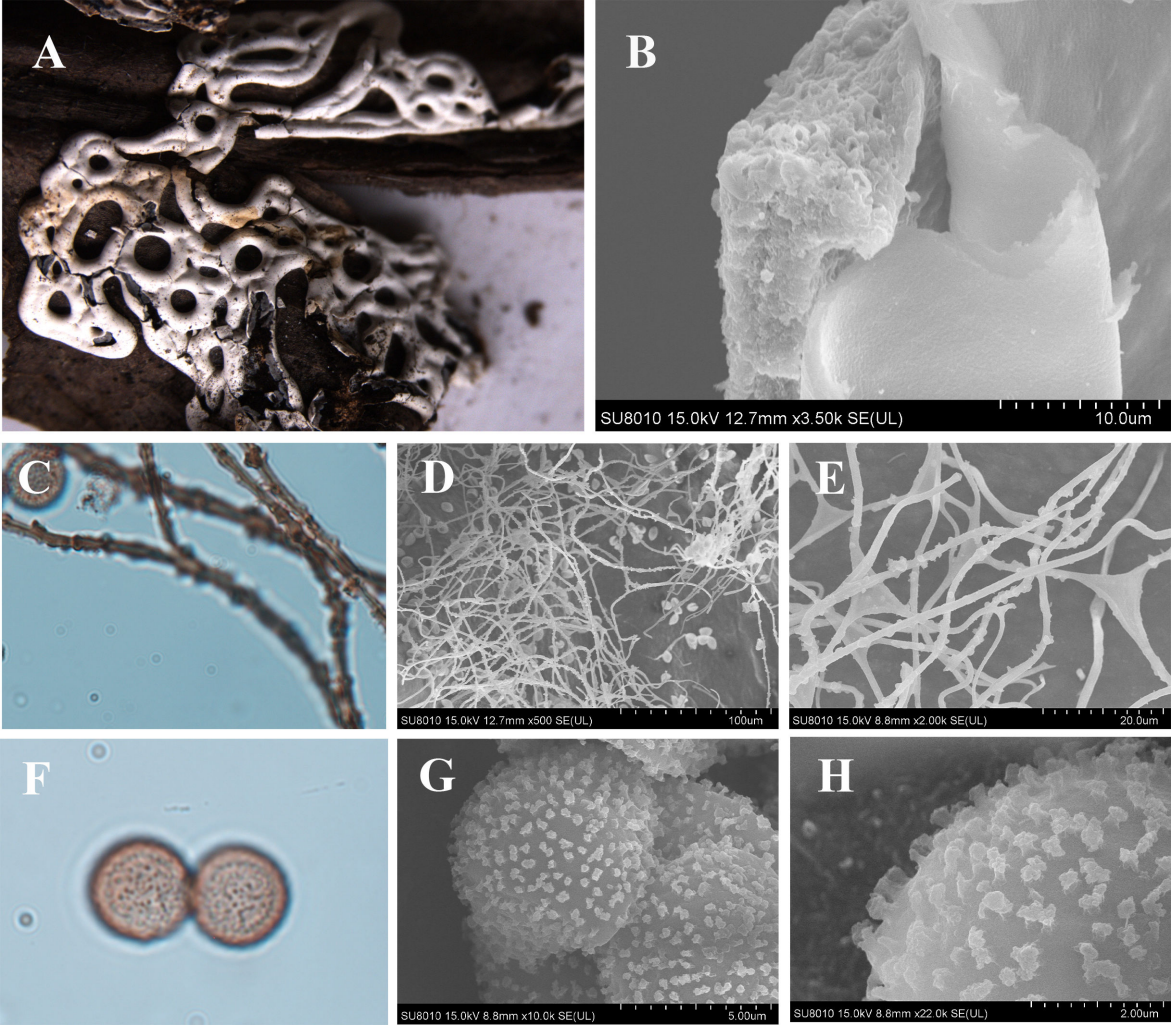
Habitat and microstructure of *D. verrucocapillitia* (HMJAU M20032). (a) Sporocarpous to plasmodiocarpous; (b) two-layered peridium by SEM; (c and d) capillitium and spores by SEM and TL; (e) capillitium with membranous enlargement and long dark spines by SEM; (f-h) spores with ridges, verrucose, and incomplete reticulation by TL and SEM. Scale bars: a = 2 mm; c = 20 µm; f = 10 µm.

***Diderma verrucocapillitia*** H. N. Zhao, B. Zhang, and Y. Li, in Zhao, Rao, Yang, Li, Yu, Zhang & Li, Phytotaxa 572 (1):66, 2022 (Fig. 17).

Description: fructification plasmodicarp, sessile, often forming branched, ring-shaped or forming a net, cushioned, white or cream Peridium two-layered, the outer layer calcareous, white, smooth, fragile, the inner layer membranous, grayish white, often tightly fused with the outer layer. Columella inconspicuous. Capillitium abundant, brown, branched, and connected, with knot and membranous enlargement. Spores dark brown in mass, pale brown by transmitted light, (8.5–) 9–10 (–11) µm in diameter, with warts connected as ridges by SEM.

Habitat: on the rotten leaves.

Distribution in China: Jilin Province.

Specimens examined: China, Jilin Province, Yanji Prefecture, Antu County, Changbai Mountain Large Sample Site, on the rotten leaves, 17 October 2017, Bo Zhang, HMJAU M20032, HMJAU M20095, HMJAU M20096, HMJAU M20097.

Notes: *D. verrucocapillitia* is often confused with *D. deplanatum* owing to morphology, they all formation of plasmodiocarps, smooth, two-layered peridium, and spores with warts. However, *D. verrucocapillitia* differs from *D. deplanatum* by its membranous extensions and dark knots in the capillitium, while *D. deplanatum* sometimes has a short stalk and an inconspicuous columella. The distribution of *D. verrucocapillitia* is relatively rare. It was reported in the Jilin Province of China in 2022 (42, 43). Here, the spores we observed are smaller than the reported spore size. Additionally, we include extra morphological characteristics of this species observed under the SEM.

***Diderma floriforme*** (Bull.) Pers., Neues Mag. Bot. 1:89, 1794.

Description: sporocarps abundant gregarious, stiptate, stout, coarse, longitudinal grooves, light grayish-brown. Peridium is two-layered, the outer layer cartilaginous, the inner layer stuck together with the outer layer, membranous, gray white. Columella conspicuous, like a bulb, light yellowish brown, rough or smooth. Capillitium is dense, slender, and straight, with many spherical and brown swellings. Spores oval or spherical, with dense spines and sparse warts, (8–) 9–10 (–11) µm in diameter.

Habitat: on the rotten woods.

Distribution in China: Gansu Province (15), Guangxi Zhuang Autonomous Region (15), Jilin Province, and Yunnan Province (15, 19).

Specimens examined: China, Jilin Province, Baishan City, Fusong County, Lushuihe Town National Forest Park Hunting Ground, on the rotten woods, 20 August 2005, Bo Zhang, HMJAU M20064, HMJAU M20122, HMJAU M20123.

Notes: *D. floriforme* shares similarities with *D. radiatum* (L.) Morgan due to its two-layered peridium (outer layer cartilaginous, inner layer membranous), dehiscent floriform below, and sporocarpic nature with a stalk or short-stalk, making *D. floriforme* similar to *D. radiatum*. However, the light grayish-brown color and non-mottled appearance in the sporocarp, long-stalk, and club-shaped columella differentiates *D. floriforme* from *D. radiatum* (15).

### Key to the species of *Diderma* present in China

1. Peridium outer layer calcareous, brittle, separated from the inner layer; sometimes the inner layer is missing, rarely petaloid dehiscent………………………………….2

2. Peridium outer layer cartilaginous, resilient, smooth, tightly fused with the inner layer, petaloid dehiscent……………………………………………………...………30

2. Peridium single-layer or appears to be single-layer, irregular dehiscent…………..3

2. Peridium obvious double-layered, irregularly, or petaloid dehiscent………………4

3. Sporocarpic chrome, ochre yellow, mars yellow, or brick-red……………………..5

3. Sporocarpic white, gray, with blue, pink, or lilac hues……………………………..6

4. Sporocarps stipitate or sessile………………………..………………………….....7

4. Sporocarps sessile ………………………………….…………………………..….8

5. Sporocarpic ochre yellow to brick-red, a layer of protuberance on the sporocarpic base, capillitium are sparse and thin…………………………………. *Diderma simplex*

5. Sporocarpic deep chrome to mars yellow. Columella absent. Capillitium is dense, with round nodules.…………………………………………………*Diderma subochraceum*

6. Peridium single-layer, sessile, pearl gray. Columella hemispherical, white. Spore diameter 8–11 μm. Grow on the dead branches or leaves. …………..*Diderma cinereum*

6. Peridium is sometimes double-layered, with or without a stalk. Grow on the moss. .…9

7. Sporocarpic discoid or hemispherical, usually with a stalk, stout…………………………………………………………*Diderma hemisphaericum*

7. Sporocarpic spherical to ovoid or inverted ovoid…………………………………10

8. Sporocarpic spherical, subspherical to pulvinate or polygonal, often densely packed or embedded in hypothallus. ……………………………………………………………..11

8. Sporocarpous to plasmodiocarpous, often flat expansion…………….……………12

9. Peridium double-layered, pearl gray, powder gray to white, with short-stalk, spores diameter 8–10 μm…………………..………………………………*Diderma montanum*

9. Peridium single layer, white or purple gray, sessile, spores diameter 10–15 μm………………………………………………………....*Diderma chondrioderma*

10. Sporocarpic pink-gray or white, columella rod-shaped, spores with warts, 10–13 μm in diameter……………….…………………………………...….. *Diderma cor-rubrum*

10. Sporocarpic white, spores with spiny, 13–13.5 μm in diameter…….*Diderma fragile*

11. Sporocarpic dense, often stacked, sometimes buried into hypothallus……….…..13

11. Sporocarpic gregarious or crowded, not buried into hypothallus…………………14

12. Sporocarpic flattened or plasmodiocarpous………………………………………15

12. Sporophores not significantly flattened…………………………………………..16

13. Sporocarpic deeply buried in developed hypothallus, outer layer peridium calcareous, coarse, tightly fused with the inner layer, spores light yellow-brown by TL, 8–11 μm in diameter………………….………………….………*Diderma spumarioides*

13. Sporocarpic not deeply buried developed or sparse hypothallus. Outer layer peridium shelly, smooth, separated from the inner layer, spores dark………..….…...17

14. Spore diameter above 12 µm……………………………………………………..18

14. Spore diameter less than 12 µm………………………………………….…….…19

15. Outer layer peridium smooth, like porcelain. Sporocarpic flat-cushion, white, lilac purple to pink, and faded to white……………………………………………………20

15. Outer layer peridium not like porcelain………………………………………..…21

16. Sporocarpic or plasmodiocarp, pulvinate or annular, white to milky white or with light purple. Columella is not well-developed and only has orange thickened on the base. …………………………..……….…..………………..……*Diderma deplanatum*

16. Sporocarpic subglobose or hemispherical, white to light pink. Columella developed…………………………..……….…………………...….…*Diderma niveum*

17. Hypothallus well-developed, spores with spiny, 11–15 μm in diameter……………………………………………………..…….*Diderma crustaceum*

17. Hypothallus not well-developed, spores with warts, 8–11 μm in diameter…………………………………………………...………...*Diderma globosum*

18. Spores polygon, with a circular and white dehiscence line, or ridge in the middle…………………………………………………..…………*Diderma cingulatum*

18. Spores globose, without light dehiscence line…………...….*Diderma liaoningensis*

19. Spores with a reticulate pattern, 2 µm in tall, 10–12 μm in diameter…………………………………..…………....….*Diderma subdictyospermum*

19. Sporea without reticulate pattern……………………………………………...…22

20. Columella hemispherical. Capillitia gray white, smooth..….….*Diderma testaceum*

20. Columella pulvinate…………………………..……….…..………...……..….…23

21. Sporocarpic, pulvinate. Capillitium without swellings and nodules. Columella pulvinate flattened…………………………..……….…..……………*Diderma effusum*

21. Plasmodiocarp. Capillitium with swellings and nodules. Columella absent or inconspicuous……………………..……….…..…………………………………….24

22. Columella is only a layer of brown protuberance on the base, or absent………..25

22. Columella pulvinate, subglobose or hemispherical, light brown to grayish white, ochre orange or yellow-brown………………………………………………………26

23. Capillitia brown, coarse, with circular nodules..............................*Diderma roseum*

23. Capillitia colorless………………………………..…………………………..…27

24. Capillitium white, spores diameter 7–8 μm……………………..*Diderma saundersii*

24. Capillitium brown, spores diameter 9–11 μm…..………..*Diderma verrucocapillitia*

25. Sporocarpic pulvinate, not stacking, ochre brown. Purple brown in the middle of the capillitium, light at both ends………………………………………*Diderma donkii*

25 . Sporocarpic hemispherical or pulvinate, often stacking, white. Capillitium colorless……………………………………………………………*Diderma gansuense*

26. Large spores, spores > 10 µm. Capillitium colorless or purple-brown…………..28

26. Small spores, spores < 10 µm. Capillitium colorless, smooth……………………29

27. Capillitium with rings.............................................................. *Diderma annuliferum*

27. Capillitium without rings, soft........................................... *Diderma flexocapillitium*

28. Sporocarps sessile. Columella pulvinate. Capillitium with dark brown swollen knots…………………………………………………………………*Diderma alpinum*

28. Sporocarps sessile or short-stalk. Sporocarpic remains as a cup-shaped substance. Columella subglobose…………………………………..………*Diderma umbilicatum*

29. Sporocarps spherical or ellipsoidal, irregular dehiscent, often broken into fragments.

……………………………………………………………………... *Diderma jilinense*

29. polygonal and cracks along the ridge, retaining a cup-shaped……*Diderma rimosum*

30. Peridium three-layered, the outer layer cartilaginous, tightly fused with the middle layer and separated from the inner layer………………………………..……………..31

30. Peridium two-layered. Sporocarpic stipitate, rarely sessile………………………32

31. Sporocarps sessile. Columella is globose or oblate, coarse, ochre, spores diameter 9–12 μm……………………………………………………………….*Diderma asteroides*

31. Sporocarps stipitate. Columella is not well-developed, white or light flesh, spores diameter 12–13 μm……………………………………………..*Diderma subasteroides*

32. Sporocarpic hemispherical to discoid, purple-brown to tea-brown, with light cracking lines and forming piebald, spores diameter 10–14 μm ……. *Diderma roanense*

32. Sporocarpic globose, subglobose, or pyriform…………………………………..33

33. Sporocarpic with pre-formed cracking line………………………*Diderma rugosum*

33. Sporocarpic without pre-formed cracking line…………………………...……..34

34. Sporocarpic light gray to light yellow-brown, with mottled or concave patterns, star-shaped or petaloid dehiscent…………………………………..……*Diderma radiatum*

34. Sporocarpic light brown to dark reddish brown, without plaques, with upper petaloid dehiscent, and lower forming cup-shaped…………………*Diderma floriforme*

## DISCUSSION

### Morphological characteristics and phylogenetic relationships of *Diderma*

The genus *Diderma* is widely distributed globally, but the number of species found in China has been relatively small. In this study, we added some new species, these newly discovered *Diderma* species exhibit unique characteristics that set them apart from previously reported species. For example, *D. roseum* has rose pink or flesh pink sporangia, and brown capillitium; while the peridium of *D. jilinense* was broken into irregular fragments, *D. gansuense* has a translucent, smooth, colorless, fewer branches of capillitium and spores with small and clumps warts. On the other hand, *D. flexocapillitium* possesses a soft capillitium, and *D. annuliferum* displays an outer layer of calcareous peridium and spherical or hemispherical sporocarps. These research findings further increase the species diversity of this genus. Phylogenetic analysis using genetic markers SSUrRNA, EF-1A, and COI, revealed that *Diderma* forms a large independent branch with three smaller branches, namely clade 1, clade 2, and clade 3, each exhibiting distinct characteristics. Our analysis showed that five species are part of clade 3. The genetic distance between these five new species was significantly greater than that of other reported sequences, aligning with our morphological observations. The characteristics of the species within clade 3 were well separated from the other two groups, characterized by a calcareous peridium, spherical or hemispherical sporocarp, and an undeveloped columella. However, some developed calcareous columellae were observed in clade 1 and clade 2. We plan to collect more specimens to further investigate this phenomenon and conduct a more detailed grouping analysis.

### Relationships within the *Didymiaceae* and *Physaraceae*

Our phylogenetic analysis uncovered significant relationships between the genus *Diderma* and the family *Didymiaceae*. Interestingly, our findings diverge from previous studies, such as those by Nandipati et al. (44), Leontyev et al. (13), and Cainelli et al. (45), which suggested a closer association between *Diderma* and the species within the family *Physaraceae*. Additionally, Fiore Donno’s research on *D. anellus* found it to be located in the *Diderma* branch, contributing to the unclear taxonomic status of *Diderma* (33). Furthermore, our examination of 11 *Physaraceae* species reveals the genera *Physarum*, *Badhamia*, and *Fuligo*. exhibit polyphyletic traits, which aligns with previous research findings (20, 23, 26, 44–46).

The genus *Didymium* also poses taxonomic complexities, as evidenced by our phylogenetic analysis, which places five selected sequences within the Stemonitidales order. Additionally, Fiore-Donno et al. suggested that Stemonitidales are paraphyletic, with Physarales emerging from within a *Lamproderma* clade. Notably, the genus *Lamproderma* itself is polyphyletic and can be subdivided into two distinct clades (33). However, definitive discussions regarding generic classification are hindered by unresolved deeper nodes in our phylogenetic tree. Additionally, the growth matrix of *Diderma* indicates that these species are mainly distributed in the northern temperate zone, with no typical tropical, East Asian, North American, or endemic types reported (12, 46–49). This suggests that *Diderma* species are not strongly associated with specific geographic regions or substrates. Given that the specimens studied mainly originate from China and there is a lack of specimens from *Diderma* and related genera in other regions, further research is warranted.

### Limitations of the present study

The study of the genus *Diderma* encountered several limitations that should be considered when interpreting the findings and planning future research. Firstly, the sampling effort may have been limited, resulting in a smaller number of specimens collected and analyzed. This could mean that the full diversity of *Diderma* species in the study areas was not adequately represented, potentially leading to incomplete insights into the genus. Additionally, the regional focus on specific provinces in China, namely Jilin, Sichuan, Liaoning, Gansu, Henan, Shaanxi, Guangdong, Hubei, and Inner Mongolia Autonomous Region, may limit the generalizability of the findings to other geographic regions or different ecological conditions where other *Diderma* and related genus species may exist. Furthermore, the taxonomic complexity of *Diderma* and related genus species presented challenges during the study. Morphological similarities among closely related species make accurate distinction difficult, potentially resulting in misidentifications or complications in characterizing specific species. Moreover, uncertainties surrounding the taxonomic status of some *Diderma* species have introduced challenges in classification and accurate identification.

The current study provides evidence of greater species diversity in *Diderma* than previously recognized. However, significant work remains. Currently, the number of known *Diderma* sequences is limited, and some sequences in the NCBI GenBank database may be incorrectly identified, which could lead to errors in species identification when using BLASTn. Therefore, a comprehensive study that integrates both morphological and molecular approaches is necessary.

Variations in morphological features used for species identification could be influenced by environmental factors or other variables, potentially leading to misinterpretations. Another limitation concerns the use of genetic markers for phylogenetic analysis. Furthermore, the lack of experimental data or functional studies may have hindered the validation of the ecological significance or functional roles of the newly identified *Diderma* and related genus species.

Lastly, ongoing discoveries of new *Diderma* species should be considered, as future research may reveal additional taxa that need to be incorporated into the study’s findings. In conclusion, while the study provided valuable insights into the diversity and relationships of *Diderma* species, it is essential to acknowledge these limitations for a comprehensive understanding and to guide future research in this field.

## MATERIALS AMD METHODS

### Sampling and morphological studies

A total of 88 specimens were collected in China. Samples collected in China over the past four decades were examined. Voucher specimens were deposited in HMJAU. After examining and describing the macroscopic characteristics of the fresh fruiting body, the sample is fixed in a specimen box for air drying in the shade and preservation.

To identify myxomycetes, the macroscopic morphological characteristics of the fruiting body under Zeiss dissecting microscope (Axio Zoom V16, Carl Zeiss Microscopy GmbH, Göttingen, Germany). Take photos using a Leica stereoscopic dissector (Leica M165, Germany). Then observe the microstructure of the fruiting body under a Zeiss light microscope (Axio Imager A2, Carl Zeiss Microscopy GmbH, Göttingen, Germany) up to 1,000×. On this basis, randomly measure the diameter of at least 20 mature spores. Photographs were taken with a Zeiss Axiocam 506 color microscope camera (Carl Zeiss Microscopy GmbH, Göttingen, Germany). Finally, the ultrastructure of spores and capillitium was observed and photographed under a SEM (Hitachi S-4800, Japan). The color terminology used is from the Flora of British Fungi: Color Identification Chart (50). The range of variation in size for sporocarps and spores is described as (minimum–)25% quartile–75% quartile(–maximum), following the latest literature standards (51).

### DNA extraction, PCR amplification, and sequencing

The total DNA from sporophores was extracted using the TIANamp Micro DNA Kit (TianGen Biotech Co., Ltd., Beijing, China) according to the manufacturer’s instructions.

We used sequences of the nSSU, EF-1α, and COI genes for phylogenetic analysis. The nSSU and EF-1α regions were amplified using the primer pairs SSU101F/P1R and PB1F/PB1R, as described by Wang et al. (52) and Novozhilov et al. (36). For COI, amplification was performed using the COMF/COMRs primer pair, which produces longer fragments. If unsuccessful, the COIF1/COIR1 primer pair was used, which produces shorter fragments, as described by Liu et al. (53), Feng & Schnittler (54), and Novozhilov et al. (55).

PCR reactions (25 µL) were prepared with 12.5 µL of 2× EasyTaq PCR SuperMix (TransGen Biotech Co., Ltd., Beijing City, China), 1 µL of each primer (10 µM), 1 µL of DNA solution, and 9.5 µL of ddH_2_O. The reaction conditions were as follows: for nSSU amplification, an initial denaturation at 95°C for 6 min, followed by 32 cycles of 95°C for 1 min, 52°C for 1 min, and 72°C for 1 min, with a final extension at 72°C for 10 min; for EF-1α amplification, an initial denaturation at 95°C for 5 min, followed by 36 cycles of 95°C for 30 s, 65.4°C for 30 s, and 72°C for 1 min, with a final extension at 72°C for 10 min (36); for COI amplification using the COMF/COMRs primer pair, an initial denaturation at 95°C for 5 min, followed by 36 cycles of 95°C for 30 s, 52°C for 20 s, and 72°C for 1 min, with a final extension at 72°C for 10 min (53, 55); for COI amplification using the COIF1/COIR1 primer pair, an initial denaturation at 95°C for 5 min, followed by 36 cycles of 95°C for 30 s, 50.7°C for 20 s, and 72°C for 1 min, with a final extension at 72°C for 10 min (54).

The PCR products were visualized under UV light after following electrophoresis on 1% agarose gels stained with ethidium bromide. Subsequently, the products were purified using the Universal DNA Purification Kit (TianGen Biotech Co., Ltd., Beijing, China). The purified PCR products were then submitted to Kumei Biotechnology Co., Ltd. (Changchun City, China) for sequencing using the Sanger method. For newly described species, two or more collections were sequenced for their nSSU, EF-1α, and COI sequences. The newly obtained sequences were deposited in GenBank (http://www.ncbi.nlm.nih.gov/genbank; Table 1).

### Data analysis

In the data analysis section, we collected and listed the sequences related to the samples based on BLAST results and morphological similarities, as presented in Table 1. The newly generated sequences were uploaded to NCBI for further analysis. We used a data set of SSU rRNA, EF-1A, and COI regions comprising sequences from this study. Moreover, we have added species from the related genera of *Diderma*, including the genus *Didymium* Schrad., *Fuligo*, *Physarum*, and *Badhamia. E. coelocephalum* and *E. minutum* were selected as the outgroup taxa.

To construct a phylogenetic tree, each gene region in the data set was first aligned using MAFFT V7.490 software (56) and manually adjusted in BioEdit v7.1.3 Sequence Alignment Editor software for accuracy (57). The best-fit evolutionary model was first estimated using Modelfinder (58). Phylogenetic analysis was then conducted with BI in PhyloSuite v1.2.2 software (59, 60). The data sets were analyzed using MrBayes 3.2.6 (61), running for 2,000,000 generations and sampling every 1,000 generations, with the initial 25% of samples discarded as burn-in. If the average standard deviation of split frequencies was above 0.01, ESS values were checked using Tracer v1.7.1 software (62), where values greater than 200 indicated convergence. For ML analyses, the data sets were examined with RaxmlGUI 2.0.5 software (63), using a rapid bootstrap with 1,000 replicates and the GTRGAMMA algorithm to find the optimal tree topology. Finally, the resulting trees were visualized with FigTree v1.3.1.

## Data Availability

The NCBI GenBank accession numbers of SSUrRNA gene sequences considered in the present study are listed in Table 1. The SSU rRNA, EF-1A, and COI gene sequencing are available from the National Center for Biotechnology Information (NCBI).

## AFTER EPUB

*[This article was published on 20 August 2024 with errors in the text, figures, legends, references, and acknowledgments. The errors were corrected in the current version, posted on 10 June 2025.]*
